# Development of an Oral Delivery System for Live Adenovirus Based on Bionic Chrysanthemum Sporopollenin Exine Armor

**DOI:** 10.3390/pharmaceutics18091107

**Published:** 2026-09-02

**Authors:** Jun Liu, Shuang Liu, Jianxiong Wei, Zifang Ding, Xiaodan Yan, Jin Sun, Shujun Wang, Shanhu Li, Yuanqing Li

**Affiliations:** 1 Wuya College of Innovation, Shenyang Pharmaceutical University, No. 103 Wenhua Road, Heping District, Shenyang 110016, China; anna_liujun@163.com (J.L.); 18840060822@139.com (Z.D.); sunjin0529@aliyun.com (J.S.); 2 National Key Laboratory of Advanced Biotechnology, Academy of Military Medical Sciences, Beijing 100071, China; shuangliu1112@163.com; 3 School of Pharmacy, Shenyang Pharmaceutical University, No. 103 Wenhua Road, Heping District, Shenyang 110016, China; xiaojianwei91@gmail.com (J.W.); wangshujun@syphu.edu.cn (S.W.); 4 School of Clinical Pharmacy, Shenyang Pharmaceutical University, No. 103 Wenhua Road, Heping District, Shenyang 110016, China; yanxiaodan07@163.com; 5 School of Traditional Chinese Medicine, Shenyang Pharmaceutical University, No. 103 Wenhua Road, Heping District, Shenyang 110016, China

**Keywords:** chrysanthemum sporopollenin, oral adenovirus vaccine, acid-shrinking/alkali-swelling, bionic enteric material

## Abstract

**Background**: The oral application of adenovirus is hindered by its poor in vitro storage stability and rapid degradation by gastric acid. To address this, a biomimetic oral adenovirus delivery system (CSP-AdV@LYO) was constructed based on three key properties of natural chrysanthemum sporopollenin (CSP): chemical inertness, intelligent “acid-shrinking/alkali-swelling” responsiveness, and mucosal adhesion via its spike structures, aiming to enhance oral stability and delivery efficiency. **Methods**: First, low-allergenic chrysanthemum pollen was screened using proteomics and a zebrafish allergy model. High-purity sporopollenin (SPO) was then extracted via an acidolysis method, followed by systematic characterization of its morphology, particle size, zeta potential, contact angle, and reversible acid-shrinking/alkali-swelling behavior. Subsequently, a CSP-AdV@LYO formulation was prepared by optimizing a cryoprotectant formulation (sucrose:gelatin = 1:1) and a vacuum loading process. Its protective and release properties were evaluated in vitro using simulated gastric and intestinal fluids, and its long-term stability was assessed. Further in vivo studies in mice assessed its intestinal colonization efficiency. The adhesion mechanism of the sporopollenin spike structures was investigated through mucosal retention experiments. **Results**: Mucosal retention experiments confirmed that the spike structures on the sporopollenin surface enhanced retention by approximately 3-fold through mechanical interlocking compared to smooth particles. In long-term stability tests, the viral genome copy number retention rate was improved more than 10-fold compared to the virus stock solution. The system enabled a steady and controlled release of the virus in simulated intestinal fluid, with the released virus maintaining its infectivity. In vivo studies demonstrated that CSP-AdV@LYO promoted efficient intestinal colonization and reduced acute mortality from 75% (AdV@LYO group) to 25%. **Conclusions**: By leveraging the unique physicochemical properties of chrysanthemum sporopollenin, this study successfully developed a biomimetic oral delivery system for live adenovirus that provides gastric acid protection, intelligent pH-responsive release, and mucosal adhesion. This system significantly enhances the oral stability and intestinal delivery efficiency of adenovirus while reducing systemic exposure risks. It offers a novel biomimetic strategy for the oral delivery of adenovirus and other biological macromolecules.

## 1. Introduction

Adenovirus (AdV) is a significant human pathogen responsible for respiratory, gastrointestinal, and ocular infections [1,2,3,4,5]. The successful deployment of live oral adenovirus vaccines, such as the licensed Ad4/Ad7 vaccine for military populations, has demonstrated both the feasibility and public health value of oral administration of live adenovirus [6,7,8]. However, the inherent biological instability of adenovirus vectors has become a major constraint on their broader application [9,10]. As a non-enveloped double-stranded DNA virus, the icosahedral capsid structure of adenovirus is highly sensitive to environmental factors such as temperature, pH, and osmotic pressure [11,12,13]. During storage and delivery, the viral capsid is prone to degradation, aggregation, or DNA leakage, leading to a rapid decline in infectious titer [14]. This challenge is particularly pronounced in the context of oral administration: the highly acidic environment and abundant proteases in the stomach can rapidly and irreversibly disrupt the integrity of viral particles, resulting in near-zero bioavailability [15]. Furthermore, the lack of efficient intestinal absorption and targeting mechanisms, along with the potential interference from pre-existing neutralizing antibodies, presents additional hurdles for effective oral delivery of live adenovirus [16]. Therefore, developing a delivery system capable of protecting adenovirus particles from degradation in the harsh gastrointestinal tract and enabling efficient intestinal retention and absorption is key to broadening the clinical application of oral adenovirus vaccines, while also offering a promising platform for the oral delivery of other biomacromolecular therapeutics [17].

Current strategies for improving adenovirus stability primarily focus on formulation engineering, such as optimizing lyophilization protectant formulations, adding stabilizers, or chemically modifying the virus surface [18]. However, these approaches often emphasize improving storage stability and still offer inadequate overall protection against the dynamic, complex challenges of oral delivery, such as extreme acidity and high enzymatic activity [19,20]. Consequently, there is an urgent need for a novel delivery platform that can provide dual protection through both physical isolation and intelligent controlled release.

Pollen, as a natural micron-scale particle, demonstrates significant potential in the field of drug delivery due to its inherent complex surface topography, good biocompatibility, and large-scale availability. However, the allergenicity induced by protein components remains a fundamental obstacle to its clinical translation [21,22,23,24]. Therefore, the selection and development of a novel carrier material that retains the advantageous natural structure of pollen while minimizing biological risks is key to overcoming bottlenecks in this field. Studies have shown that pollen allergens are primarily associated with certain soluble protein components, while the pollen exine, sporopollenin, is an exceptionally chemically inert biomacromolecule [25,26,27]. The removal of internal cytoplasm, the intine, and soluble proteins attached to the exine surface via physicochemical methods yields hollow microshells primarily composed of sporopollenin, known as pollen exine shells. This process theoretically eliminates the major proteinaceous allergens, offering the potential to convert highly allergenic natural pollen into a low-allergenicity biomaterial [28,29].

Previous research has shown that sporopollenin, the primary skeletal component of the pollen exine, is a complex biopolymer formed by the highly cross-linked polymerization of carotenoids and phenolic lipids. It is remarkably resistant to degradation by strong acids, alkalis, organic solvents, high temperatures, and most biological enzymes [30,31]. Chrysanthemum sporopollenin exhibits a unique “acid-shrinking/alkali-swelling” pH-responsive behavior: in the acidic gastric environment (pH ~2.0), its germination pores contract, particle size decreases, and the surface becomes hydrophobic (contact angle > 120°); in the near-neutral intestinal environment (pH 7.0–10.0), the pores expand, particle size increases by approximately 30%, and the surface becomes hydrophilic (contact angle < 30°), with this response being reversible [32]. Furthermore, to meet the clinical requirements for an orally administerable, non-viral, and capable of surviving in harsh gastrointestinal environments for the delivery of adenovirus, Chrysanthemum sporopollenin has significant advantages. Firstly, the natural presence of phenolic groups in Chrysanthemum sporopollenin is higher, resulting in stronger acid resistance and antioxidant capacity. Secondly, the densely distributed spike-like nanostructures on the surface of Chrysanthemum sporopollenin can form mechanical interlocking with the intestinal mucus layer, significantly enhancing mucosal retention. Finally, compared to other plant pollen, its immunogenicity is lower [33,34].

Leveraging these core properties, this study proposes a tripartite biomimetic delivery strategy integrating “exine armor protection, intelligent enteric release, and spike-mediated adhesion to construct an oral delivery system for live adenovirus”: (1) Utilizing the physical barrier effect of the sporopollenin cavity to enhance the in vitro storage stability of adenovirus; (2) Employing the chemically inert skeleton and “acid-shrinking” behavior to form a tight protective armor in the stomach; (3) Exploiting the “alkali-swelling” behavior to open germination pores for targeted release in the intestine; (4) Utilizing the mechanical interlocking effect of the spike structures to prolong the duration of intestinal mucosal retention. This research systematically validates the effectiveness of this delivery system, from carrier screening and formulation development to in vitro and in vivo evaluation.

## 2. Materials and Methods

### 2.1. Materials

Instruments: SHZ-D III Circulating Water Vacuum Pump (Yuhua Instrument Co., Ltd., Suzhou, China), DF-101S Constant Temperature Heating Magnetic Stirrer (Yuhua Instrument Co., Ltd., Suzhou, China), LGJ-10F Lyophilizer (Beijing Songyuan Huaxing Technology Development Co., Ltd., Beijing, China), PX85Z Analytical Balance (Ohaus Instrument Co., Ltd., Parsippany, NJ, USA), FJ200-SH Digital High-Speed Dispersing Homogenizer (Shanghai Specimen & Model Factory, Shanghai, China), SCIENCETZ-E Ultrasonic Cell Disruptor (Ningbo Scientz Biotechnology Co., Ltd., Ningbo, China), TDZ4K Centrifuge (Hunan Xiangyi Laboratory Instrument Development Co., Ltd., Changsha, China), HY-2 Low-Speed Multifunction Oscillator (Guohua Electrical Appliance Co., Ltd., Changzhou, China), Ultimate 3000 Capillary High-Performance Liquid Chromatograph (Thermo Fisher Scientific, Waltham, MA, USA), ESI Combined Ion Trap Orbitrap Mass Spectrometer (Thermo Fisher Scientific, Waltham, MA, USA), XD-202 Optical Microscope (Caikang Optical Instrument Co., Ltd., Shanghai, China), S-3400N Scanning Electron Microscope (Hitachi, Tokyo, Japan), MX-S Adjustable Vortex Mixer (Dragon Lab Instruments Co., Ltd., Beijing, China), HELOS/KF Dry/Wet Particle Size Analyzer (Sympatec GmbH, Clausthal-Zellerfeld, Germany), UV-Vis Spectrophotometer (Shanghai Yuanxi Instrument, Shanghai, China), ZS90 Malvern Particle Size Analyzer (Malvern Instruments Ltd., Worcestershire, UK), and DSA25 Contact Angle Analyzer (KRÜSS GmbH, Hamburg, Germany).

Reagents: BAPNA (Shanghai Yuanye Bio-Technology Co., Ltd., Shanghai, China), Compound 48/80 (Shanghai Yuanye Bio-Technology Co., Ltd., Shanghai, China), Iodoacetamide (Sigma-Aldrich, St. Louis, MO, USA), Trypsin (Promega, Madison, WI, USA), Urea (Thermo Fisher Scientific, Waltham, MA, USA), Ammonium Bicarbonate (Anhui Zesheng Technology Co., Ltd., Anqing, China), BCA Protein Assay Kit (Beyotime Biotechnology, Shanghai, China), Formic Acid (Thermo Fisher Scientific, Waltham, MA, USA), Methanol (Thermo Fisher Scientific, Waltham, MA, USA; Anhui Shilian Special Solvent Co., Ltd., Anqing, China), Acetonitrile (Thermo Fisher Scientific, Waltham, MA, USA), Purified Water (Hangzhou Wahaha Group Co., Ltd., Hangzhou, China), Methylene Blue (Beijing Solarbio Science & Technology Co., Ltd., Beijing, China), Phosphoric Acid (Chengdu Kelong Chemical Reagent Co., Ltd., Chengdu, China), Hydrochloric Acid (Tianjin Fuyu Chemical Reagent Factory, Tianjin, China), Ethanol (Tianjin Fuyu Chemical Reagent Factory, Tianjin, China), Acetone (Tianjin Fuyu Chemical Reagent Factory, Tianjin, China), Sodium Hydroxide (Tianjin Fuyu Chemical Reagent Factory, Tianjin, China), chrysanthemum Pollen (North Tianshan, Xinjiang, China), Sunflower Pollen (Bayannur, Inner Mongolia, China), Rape Pollen (Haixi, Qinghai, China), Amur Cork Tree Pollen (Bayannur, Inner Mongolia, China), Masson pine Pollen (Songshan Township, Shandong, China), Jujube Pollen (Aksu, Xinjiang, China), Japanese Climbing Fern Spore (North Tianshan, Xinjiang, China), Mucin (Shanghai Aladdin Biochemical Technology Co., Ltd., Shanghai, China), Agarose Gel (Beijing Solarbio Science & Technology Co., Ltd., Beijing, China), Glycine (Hebei Chuangshou Biotechnology Co., Ltd., Handan, China), Absolute Ethanol (Fuyu Fine Chemical Co., Ltd., Fuyu, China), Concentrated Hydrochloric Acid (Fuyu Fine Chemical Co., Ltd., Fuyu, China), Indocyanine Green (Prepared in-house), Phosphate-Buffered Saline (Tianjin Haoyang Huake Biotechnology Co., Ltd., Tianjin, China), Adenovirus (Wujiahe Gene Technology Co., Ltd., Beijing, China), Sucrose (Shanghai Aladdin Biochemical Technology Co., Ltd., Shanghai, China), Gelatin (Gelita (Liaoyuan) Gelatin Co., Ltd., Liaoyuan, China), Soybean Lecithin (Nanjing Well Pharmaceutical Group Co., Ltd., Nanjing, China), and Chrysanthemum Sporopollenin (Prepared in-house). BCA Protein Assay Kit (Beyotime Biotechnology, Shanghai, China). HAdV-4 was obtained from the American Type Culture Collection (ATCC) and propagated in our laboratory. All operations involving live HAdV-4 and related mouse experiments were conducted in an Animal Biosafety Level 2 (ABSL-2) facility. Virus purification was completed by Shenzhen Hanmei Biotechnology Co., Ltd., Shenzhen, China.

Animals and Cell Lines: Adult wild-type AB strain zebrafish (3–4 cm) were purchased from the China Zebrafish Resource Center. SPF grade hCXADR-humanized C57BL/6JNifdc mice (expressing human CXADR, the receptor for adenovirus) were purchased from Shanghai Model Organisms Center, Inc. SPF grade BALB/c mice (female, 6–8 weeks) were purchased from Beijing Vital River Laboratory Animal Technology Co., Ltd. All mice were maintained under specific pathogen-free (SPF) conditions in a temperature-controlled facility (22 ± 2 °C) with a relative humidity of 50–60%. The animals were housed under a strict 12 h light/dark cycle (lights on at 07:00), with ad libitum access to sterilized food and autoclaved water. All experimental procedures were performed in accordance with the institutional guidelines for animal care and use. HEK293 cells were purchased from Zhejiang Meisen Cell Technology Co., Ltd., Jinhua, China.

### 2.2. Protein Extraction from Pollen

Extraction of surface-associated proteins from Pollen: Precisely 0.50 g of defatted sunflower, chrysanthemum, rape, masson pine, jujube, Ganoderma lucidum, cattail pollen, and Japanese climbing fern spore samples, respectively, were weighed and placed into separate 50 mL centrifuge tubes. To each tube, 10 mL of an alkaline extraction buffer (pH 10.0) was added. The mixtures were subjected to sonication in an ice-water bath for 30 min at room temperature. The sonication power was set to 300 W using a pulsed mode (2 s on, 3 s off). This process aimed to dissociate proteins loosely bound to the pollen exine or adherent to the sporopollenin matrix without compromising the structural integrity of the pollen wall. Following treatment, the mixtures were vacuum-filtered through a 0.45 µm mixed cellulose ester membrane, and the resulting filtrate was collected as the surface protein extract. The pollen particles retained on the membrane were transferred to a vacuum desiccator and dried overnight at room temperature to obtain dried pollen residue.

Extraction of Internal Proteins from Pollen: The aforementioned dried pollen residue samples were transferred to a pre-cooled mortar, rapidly frozen with a small amount of liquid nitrogen, and then ground continuously for 10 min using a pestle. The ground pollen powder was quantitatively transferred to 15 mL centrifuge tubes, and 10 mL of pre-cooled alkaline extraction buffer (pH 10.0) was added. The suspensions were sonicated in an ice-water bath for 30 min. The resulting homogenates were vacuum-filtered through a 0.45 µm membrane, and the collected filtrate constituted the internal protein extract.

### 2.3. Proteomic Analysis of Pollen

Protein concentration was determined quantitatively using the BCA method, and the concentration of all samples was normalized based on the results. Subsequent reduction and alkylation were performed as follows: 100 µL of 10 mM dithiothreitol (DTT, prepared in 50 mM NH_4_HCO_3_) was added to the samples, followed by incubation with shaking at 56 °C for 30 min. The supernatant was then discarded, and 100 µL of 55 mM iodoacetamide (IAA, prepared in 50 mM NH_4_HCO_3_) was added for alkylation, with the reaction proceeding for 1 h at room temperature in the dark. After discarding the supernatant, 50 µL of trypsin (Trypsin) was added for enzymatic digestion with shaking at 37 °C for 16 h. The reaction was terminated by adding 10 µL of formic acid (final concentration 1%). The supernatant was transferred to a new tube, and a 5 min sonication-assisted extraction was performed. The supernatants from the two steps were combined. Desalting was carried out using C18 ZipTips (OmicSolution Biotechnology Co., Ltd., Shanghai, China), and the resulting peptide solution was vacuum-concentrated to near dryness. The peptides were then reconstituted in 50 µL of 0.1% formic acid in purified water for subsequent mass spectrometric analysis.

Raw mass spectrometry data were searched against a database using the Sequest HT algorithm. The obtained protein sequences were imported into the AllerCatPro platform for potential allergen prediction analysis. During data processing, key parameters in the AllerCatPro output were systematically screened. Allergenicity Classification: Protein sequences were submitted to the AllerCatPro 2.0 server (https://allercatpro.bii.a-star.edu.sg/ (accessed on 4 April 2026)) for in silicoallergenicity risk assessment. The classification into “strongly” or “weakly” allergen-associated was based on a composite scoring system integrating: (i) full-length sequence identity (≥50% over ≥80% length); (ii) 80-aa sliding window identity (≥30%); (iii) 3D structural homology; and (iv) 6-mer peptide motif matching. Proteins designated as “strongly allergen-associated” met the criteria for “High similarity to known allergens” and contained significant 6-mer epitopes or repetitive motifs (e.g., Q-repeats). Proteins classified as “weakly allergen-associated” exhibited low-to-moderate sequence similarity without high-confidence epitope signatures. Results were cross-validated against the WHO/IUIS Allergen Nomenclature Database. Proteins yielding “no hits” were annotated to distinguish potentially low-allergen-risk proteins.

### 2.4. Zebrafish Allergy Assay

Adult wild-type AB zebrafish (Danio rerio), 3–4 cm in length (4–6 months post-fertilization), of mixed sex, were randomly assigned to groups of 6 fish each. The assay was an exploratory acute screening model. Group size was selected based on established zebrafish allergology protocols. Negative Control Group: reaction buffer (0.1 M Tris-HCl, pH 8.2) with 250 μg/mL BAPNA (Nα-benzoyl-DL-arginine 4-nitroanilide hydrochloride, Sigma-Aldrich Cat# B4875) substrate solution. Positive controls included 10 μg/mL Compound 48/80 (Sigma-Aldrich Cat# C2313). Pollen test groups contained 100 μg/mL of the respective pollen extract in the BAPNA buffer. Each zebrafish was placed individually in a 5 mL EP tube containing 2 mL of the corresponding reaction solution. The tubes were incubated at 25 °C under constant temperature for 1 h in the dark. After incubation, the reaction solution was transferred to a new centrifuge tube and centrifuged at 4 °C and 5000 rpm for 10 min. The supernatant was collected as the test sample for tryptase activity measurement. The absorbance of p-NA (hydrolysis product of substrate BAPNA) in the resulting supernatant samples was measured at 405 nm using a microplate reader. Each sample was measured in triplicate.

A calibration curve was established using p-nitroaniline (p-NA, Sigma-Aldrich Cat# N8630) at concentrations ranging from 0 to 100 μM in the same buffer. Absorbance was measured at 405 nm in triplicate. Tryptase-like activity was quantified by interpolation against the p-NA standard curve and normalized to total protein content (measured by BCA assay), expressed as nmol p-NA released/min/mg protein.

### 2.5. Preparation of Pollen Sporopollenin

The extraction and purification of sporopollenin were performed with modifications based on the classic method involving acid hydrolysis combined with multi-step solvent washing. The detailed procedure is as follows: Precisely 5.0 g of each pollen type was weighed separately and dispersed in 50 mL of deionized water. The mixture was filtered through an 80-mesh sieve to remove macroscopic impurities. The residue was then subjected to vacuum filtration and repeatedly washed until the filtrate became clear and colorless. The pollen was subsequently washed sequentially with 50% ethanol, 75% ethanol, and absolute ethanol until the filtrate was colorless. The sample was dried in a 40 °C oven for 4 h. Next, it was suspended in 85% (*v*/*v*) phosphoric acid and reacted under continuous stirring in a 50 °C oil bath for 6 h. The intermediate product was obtained via vacuum filtration. A series of rigorous and ordered surface activation washes followed: The product was first washed with 50 mL of hot deionized water until the filtrate was neutral, then treated with 50 mL of hot 2 M sodium hydroxide solution, and subsequently washed again with hot water to neutrality. Thereafter, it was washed sequentially with 50 mL of hot acetone, 50 mL of hot 2 M hydrochloric acid, 50 mL of hot deionized water (to neutrality), 50 mL of hot acetone, 50 mL of hot ethanol, and finally 50 mL of hot deionized water. The contact time between the solid and each solvent was controlled at 5 min per step. Upon completion of all washing steps, the final product was collected by vacuum filtration, spread evenly on a clean glass culture dish, and dried in a 40 °C oven for 4 h. The resulting solid was the purified pollen sporopollenin, which was stored in a desiccator for later use.

### 2.6. Elemental Analysis

The sporopollenin samples from various sources were subjected to detection and analysis using an elemental analyzer to determine the content of nitrogen (N), phosphorus (P), and sulfur (S) elements. The protein content in sporopollenin was estimated based on the measured percentage of N, using a multiplication factor of 6.25, which may overestimate true protein levels in sporopollenin; thus, these values should be interpreted as relative indicators.

### 2.7. Scanning Electron Microscopy (SEM) Analysis

A small amount of dried sporopollenin powder was evenly dispersed onto a specimen stub pre-fitted with conductive double-sided adhesive tape. Loosely adhered particles were gently removed using a rubber air blower. To enhance sample conductivity, a platinum layer approximately 10 nm thick was sputter-coated onto the sample surface using an ion sputter coater. The prepared specimen stub was then placed in the SEM sample chamber. Representative area images were observed and captured in secondary electron imaging mode at an accelerating voltage of 5–10 kV and an appropriate working distance. This allowed for the analysis of particle size, shape, surface texture, and multi-layered structural features.

### 2.8. Macrophage Immunoassay Using Neutral Red Staining

Neutral red staining was employed to quantitatively analyze macrophages in zebrafish. Zebrafish embryos were first placed in appropriately sized containers. Upon reaching 24 h post-fertilization (hpf), 0.003% 1-phenyl-2-thiourea (PTU) was added. A negative control group and different pollen component sensitization groups (final concentration: 100 μg/mL) were established, with 15 embryos per group and three parallel experiments for each. At 48 hpf, the corresponding pollen components were added to the intervention groups to induce an inflammatory response, which continued until 72 hpf. After 72 hpf, neutral red staining solution (10 μg/mL, containing 0.003% PTU) was added to the embryo culture medium of each group. The embryos were incubated in the dark within a constant-temperature incubator for 2.5 h to stain the macrophages. Subsequently, the larvae were repeatedly washed until the wash solution became clear and were then anesthetized using 0.003% tricaine methanesulfonate (MS-222). The caudal hematopoietic tissue (CHT) region of the tail was observed and imaged under an optical microscope to examine macrophage recruitment. The number of recruited macrophages was counted, manual counting of macrophages within the CHT region was performed on static images by two independent, blinded investigators, the cell counts from each sensitization group were compared to assess allergenicity.

### 2.9. Neutrophil Immunoassay Using Sudan Black B Staining

This study utilized Sudan Black B staining to quantitatively detect neutrophil recruitment in zebrafish. Zebrafish were grouped and subjected to drug intervention as described in Section 2.7. Successfully incubated zebrafish were then stained with Sudan Black B. The zebrafish were placed in PBS at room temperature and fixed with 4% paraformaldehyde (PFA) solution for 10 h. After fixation, the zebrafish were rinsed in PBS and incubated in freshly prepared Sudan Black B solution in the dark for 2 h to stain neutrophils. The larvae were then washed with 75% ethanol until the wash solution became clear and anesthetized with 0.003% MS-222. Neutrophil aggregation in the tail region of the larvae was observed under a microscope, and the neutrophil count was determined to assess allergenicity.

### 2.10. Particle Size Analysis

Sporopollenin powders were dispersed in deionized water to prepare suspensions at appropriate concentrations. The suspensions were sonicated for 5 min to ensure homogeneity. Particle size analysis was performed using a Malvern laser particle size analyzer at a constant temperature of 25 °C. Prior to measurement, a background scan with pure water was conducted to ensure compliance with background signal requirements. The sample cell was then filled with an adequate amount of the suspension. After the system temperature equilibrated, the measurement was initiated. Each sample was measured automatically three times consecutively, with a data acquisition time of 60 s per measurement. Triplicate independent measurements of particle sizes (*n* = 3) were performed at each pH condition.

### 2.11. Zeta Potential Analysis

Sample powders or concentrated dispersions were diluted to an appropriate concentration with ultrapure water. The diluted samples were carefully pipetted into a dedicated folded capillary Zeta potential cell, avoiding the introduction of air bubbles. The cell was then inserted into the sample holder of the Malvern particle size analyzer. The measurement temperature was set to 25 °C, and the system was allowed to equilibrate for 120 s to achieve temperature stability. Following equilibration, the automatic measurement program was started. Each sample underwent three consecutive measurements, with each measurement consisting of 20 sub-measurements. The instrument automatically calculated the average Zeta potential and standard deviation.

### 2.12. Morphological Observation Under Optical Microscope

Sporopollenin powders were dispersed in solutions with pH values of 2.0, 7.0, and 10.0, respectively. A small drop of each suspension was placed on a clean microscope slide, and a coverslip was gently placed on top and pressed lightly to form a thin, uniform monolayer. The prepared specimens were observed under an optical microscope using a 100× oil immersion objective lens. The micromorphology of the sporopollenin microcapsules was examined, and digital images were captured for documentation.

### 2.13. Contact Angle Measurement

To characterize the changes in the wettability of the sporopollenin surface with environmental pH, the sessile drop method was employed for quantitative assessment. 0.50 g of sporopollenin powder was placed on the sample stage and compacted using a clean glass slide or a dedicated tablet press to form a flat, dense, and uniformly thick test surface. Test liquids with pH 2.0, pH 7.0, and pH 10.0 were prepared. In a 25 °C environment, a 2.0 µL droplet of the test liquid was dispensed onto the sample surface. An image of the droplet was captured immediately upon contact with the surface. The static contact angles were calculated by analyzing the tangent angle at the solid–liquid–gas three-phase contact point in the image and triplicate independent measurements (*n* = 3) were performed at each pH condition.

### 2.14. In Vitro Mucoadhesion Assessment of Sporopollenin

A biomimetic mucus matrix was constructed to evaluate the in vitro mucoadhesive properties of the drug-loaded sporopollenin system. Firstly, the biomimetic mucosal environment was prepared: 5% mucin was uniformly mixed with agarose gel in phosphate-buffered saline, heated and dissolved in a 60 °C water bath, and then poured onto a glass plate. The mixture was cooled and solidified at room temperature to form the biomimetic mucosal layer. The solidified biomimetic mucosal sheet was fixed at a slight incline and equilibrated at 37 °C. Subsequently, 100 µL aliquots of a ICG-loaded sporopollenin suspension and a ICG solution were evenly applied onto the surface of the biomimetic mucosa, respectively. Simulated physiological saline was then perfused gently over the mucosal surface at a rate of 0.1 mL/min using a peristaltic pump to mimic the physiological clearance process of mucus in vivo. The perfusate was continuously collected. At time points of 5, 10, 15, 20, 30, 60, and 120 min, the absorbance intensity of the collected perfusate was measured using a UV-Vis spectrophotometer. The cumulative retention rate of the delivery system on the biomimetic mucosal surface was calculated.

### 2.15. In Vivo Mucoadhesion Assessment of Sporopollenin

Mice were divided into a negative control group and a sporopollenin group. Anesthetized via isoflurane inhalation, mice in the sporopollenin group were orally administered 200 µL of an indocyanine green (ICG)-labeled chrysanthemum sporopollenin suspension, while the negative control group received 200 µL of an ICG solution via oral gavage, with 3 mice per group. At different time points (0 h, 2 h, 6 h, 12 h, 24 h), the mice were imaged using an in vivo imaging system. The images were recorded to compare the retention duration of the different formulations within the mouse gastrointestinal tract.

### 2.16. Drug Loading Methods and Determination of Drug Loading Capacity

Precisely 10.0 mg of sporopollenin was weighed and mixed with 5.0 mL of a 0.5 mg/mL methylene blue aqueous solution. Three different loading methods were then employed: Ultrasonic Loading Group: The mixture was subjected to pulsed ultrasonic treatment at 250 W power in an ice bath for 30 min. Vortex Loading Group: The mixture was continuously vortexed at 3000 rpm for 30 min. Vacuum Loading Group: The mixture was placed in a vacuum desiccator and subjected to negative pressure for 30 min. After the loading process, the mixtures were filtered to separate the unloaded drug solution. The solid precipitate was repeatedly washed with deionized water. The obtained solid was then resuspended in 3.0 mL of deionized water and centrifuged at 4 °C and 10,000 rpm for 10 min. The absorbance of the supernatant was measured at 664 nm using UV-Vis spectrophotometry. The drug loading capacity per unit mass of sporopollenin was calculated.

### 2.17. Optimization of Drug Loading Conditions: Effects of Temperature, pH, and Surfactant

Using the predetermined optimal drug loading method, the effects of temperature, surfactant, and pH on the drug loading capacity were investigated separately. For all experiments, the sporopollenin mass was fixed at 10.0 mg, the methylene blue solution concentration at 0.5 mg/mL, and the reaction system volume at 5.0 mL. The specific procedures were as follows: For the temperature effect study, five gradients (10, 25, 30, 37, and 60 °C) were set. The sporopollenin-drug mixture was incubated in the dark with shaking for 2 h in a constant-temperature water bath shaker set to the corresponding temperatures. The surfactant effect was investigated by comparing groups with and without the addition of 0.1% (*w*/*v*) Tween 80. For the pH effect study, methylene blue solutions were prepared using phosphate-buffered saline (pH 2.0, 4.0, 7.0) and glycine-NaOH buffer (pH 8.0, 10.0, 13.0) for drug loading. After incubation, the drug-loaded sporopollenin from each experimental group was collected by filtration, resuspended in 5.0 mL of deionized water, and subjected to high-speed centrifugation. The supernatant was collected, and the residual drug concentration was determined at 664 nm using UV-Vis spectrophotometry. The adsorption amount per unit mass of sporopollenin under different conditions was calculated. Each group was tested in triplicate.

### 2.18. Screening of Freeze-Drying Protectant Formulations

To screen and optimize the freeze-drying protectant formulation for adenovirus, this study systematically compared the effects of different protectant compositions (sucrose, gelatin, phospholipid) on the physical characteristics, moisture content, and viral genome copy number retention rate of the lyophilized product [35,36]. The formulations tested are shown in Table 1 below.

### 2.19. Investigation of Sporopollenin Dosage

To optimize the loading efficiency and protective performance of sporopollenin as a natural microcarrier in the lyophilized adenovirus formulation, its dosage is a critical process parameter. Given the presence of a composite freeze-drying protectant system, the vacuum loading method used previously is not suitable for the loading process in this context. Therefore, this study systematically optimized the sporopollenin dosage through an oscillation adsorption experiment, using the model dye methylene blue as an indicator. Precisely weighed sporopollenin samples of varying masses were each mixed with a known concentration of 0.1 mM methylene blue solution in sealed containers. The mixtures were incubated with constant shaking in a 25 °C thermostatic shaker until adsorption reached dynamic equilibrium. Subsequently, the mixtures were filtered, and the filtrate was collected. The absorbance of the filtrate was measured at 664 nm using a UV-Vis spectrophotometer. The residual concentration of the dye in the solution was calculated based on a pre-established methylene blue standard curve. By comparing the concentration difference in methylene blue in the solution before and after adsorption, the adsorption capacity of methylene blue per unit mass of sporopollenin was calculated. A relationship curve between the adsorption capacity and the sporopollenin dosage was then plotted. The saturation point or plateau region corresponding to maximum adsorption efficiency was determined, and the sporopollenin dosage at this point was defined as the optimal carrier input for subsequent virus loading experiments.

### 2.20. Preparation of AdV@LYO, CSP-AdV@LYO, and CSP-PL-AdV@LYO

Under continuous gentle stirring, pre-cooled adenovirus stock was slowly added to a sucrose solution to obtain a homogeneous adenovirus-sucrose mixture. A prescribed amount of gelatin powder was weighed, slowly added to cold water for injection, and allowed to swell for 30 min. The swollen system was then placed in a 50 °C water bath with continuous stirring until the gelatin was completely dissolved, forming a clear gelatin solution. The prepared adenovirus-sucrose mixture and the gelatin solution were combined and stirred for 20 min to obtain a milky, homogeneous liquid, which served as the adenovirus solution to be lyophilized (AdV@LYO precursor).

AdV@LYO: The aforementioned adenovirus solution (AdV@LYO precursor) was subjected to lyophilization. Under aseptic conditions, it was quantitatively aliquoted into pre-treated sterile vials. The pre-freezing stage employed a programmed cooling method: cooling from room temperature to 0 °C at a rate of 1 °C/min, holding for 1 h, then cooling to −50 °C at 1.5 °C/min and maintaining for 2 h. Primary drying was conducted at −5 °C under a vacuum for 34 h. For desorption drying, the temperature was gradually increased to 20 °C, and drying continued for 8 h to reduce the residual moisture content to less than 3%.

CSP-AdV@LYO: A weighed amount of sporopollenin (CSP) sample was mixed with the AdV@LYO precursor solution. The mixture was placed in a 25 °C constant temperature shaker and incubated with shaking at 150 rpm for 4 h. The resulting sporopollenin-virus complex suspension was quantitatively aliquoted into vials under aseptic conditions and immediately transferred to the freeze-dryer. It was then dried according to the lyophilization process described above, ultimately yielding CSP-AdV@LYO.

CSP-PL-AdV@LYO: Following virus loading onto the CSP, a phospholipid membrane was deposited onto its surface. Soybean lecithin (Soy PC) and cholesterol (Chol) were combined at a mass ratio of 9:1 and dissolved in anhydrous ethanol to a final total lipid concentration of 10 mg/mL, which was sterile-filtered through a 0.22 μm membrane to remove bacterial contaminants. The sporopollenin-virus complex prepared above was dispersed in this phospholipid-ethanol solution to form a uniform suspension (lipid-to-sporopollenin ratio: 3:1 *w*/*w*). This suspension was then transferred to a rotary evaporator. Rotary evaporation was performed in a 40 °C water bath to allow uniform deposition of the phospholipids onto the carrier surface, forming a continuous biomimetic membrane. After the solvent was completely removed, a biomimetic sporopollenin intermediate coated with a phospholipid membrane was obtained. 20 mg of the CSP–phospholipid complex were washed three times with 0.5 mL of sterile, phosphorus-free water (4 °C) to remove non-adherent lipid. The solid was sedimented by centrifugation at 3000× *g* for 5 min after each wash, and the combined supernatants were collected as the free lipid fraction (M_free_). A known aliquot of the original ethanolic phospholipid solution (total lipid M_total_) was taken as the input control. Both fractions were digested with 70% perchloric acid at 180 °C until colorless, neutralized, and reacted with ammonium molybdate-ascorbic acid reagent; absorbance was read at 830 nm against a KH_2_PO_4_ standard curve. Coating efficiency was defined as (M_total_ − M_free_)/M_total_ × 100% and determined as 90.2 ± 2.8% (*n* = 3). This intermediate was then subjected to freeze-drying according to the aforementioned lyophilization process, ultimately producing CSP-PL-AdV@LYO.

### 2.21. Flowability Assessment

The angle of repose of the enteric particles was determined using the fixed funnel method in a temperature- and humidity-controlled room (25 ± 1 °C, 50 ± 5% relative humidity). A clean, dry glass funnel was fixed on a retort stand, with its lower outlet positioned 5 cm above the surface of a glass culture dish. Then, 5 g of the particulate sample was poured into the center of the funnel. After the formed pile stabilized, the angle between the inclined surface of the pile and the horizontal plane was measured using a protractor to obtain the angle of repose of the lyophilized particles. Each sample was measured in triplicate.

### 2.22. Stability Test of Indocyanine Green Under pH Changes

Samples were immersed in a pH 2.0 solution for 6 h. Subsequently, the solution pH was adjusted to 7.4, and the drug was rapidly released via sonication. The absorption peak at 784 nm was examined using a UV-Vis spectrophotometer to quantify the content of ICG.

### 2.23. Release Test of Indocyanine Green in Simulated Intestinal Fluid

A dissolution test was conducted in simulated intestinal fluid (10 mM NaHCO_3_, 120 mM NaCl, 3.5 mM KCl, 0.5 mM MgSO_4_, 0.7 mM CaCl_2_ and 200 U/mL pancreatin). Samples were precisely collected at time points of 0, 5, 10, 15, 20, 30, and 60 min. The absorption peak at 784 nm was examined using a UV-Vis spectrophotometer to quantify the content of ICG.

### 2.24. Virus Stability Test

A dissolution test was conducted. Samples were precisely collected at time points of 1, 3, 6, 9, and 12 h. The collected samples were immediately filtered through a 0.45 µm membrane to obtain the released virus solution at each time point. Subsequently, each release solution sample was used to infect a susceptible cell line. After continuous culture for 6 days, the cells were harvested and subjected to three freeze–thaw cycles to release intracellular progeny viruses. Viral genomic DNA was then extracted and absolute quantification was performed via qPCR, with the viral genome copy number calculated based on a standard curve to confirm the infectivity of the released adenovirus.

### 2.25. Stability Testing of Formulations

To evaluate the stability trend of the formulations under non-recommended long-term storage conditions, an long-term stability test was conducted. Because adenovirus stocks are conventionally stored at −80 °C, the 2–8 °C/75% RH condition used here represents a deliberate suboptimal challenge serves as an accelerated-like stress test for the delivery system. The three types of samples were stored under long-term testing conditions. Samples were taken at preset time points: initial, and at 1 and 3 months. A comprehensive analysis of the samples’ appearance and viral genome copy number was performed. The stability of the formulations’ physicochemical properties and biological activity was assessed by analyzing the change trends of these indicators over time.

### 2.26. Toxicity Test

Free adenovirus suspension prepared in sterile PBS served as a negative control, and a parallel experiment was conducted with the three lyophilized formulations: AdV@LYO, CSP-AdV@LYO, and CSP-PL-AdV@LYO. Healthy mice were randomly divided into four groups (*n* = 4) and orally administered the respective formulation or control, the administration dose was 2 × 10^4^ viral copies per mouse. The animals were continuously monitored for 24 h post-administration. During this period, survival, clinical signs, behavioral activity, and general condition were systematically recorded for all groups. Using 24 h post-administration as the observation endpoint, the cumulative survival rates of animals in each group were calculated and compared.

### 2.27. In Vivo Efficacy Study

To systematically evaluate the in vivo stability, biodistribution, and targeted delivery characteristics of the three formulations, a mouse study was conducted. Free adenovirus suspension prepared in sterile PBS served as a negative control, and a parallel comparison was made among the three lyophilized formulations: AdV@LYO, CSP-AdV@LYO, and CSP-PL-AdV@LYO. The administration dose was 2 × 10^4^ viral copies per mouse. Healthy hCXADR-humanized C57BL/6JNifdc mice were randomly assigned to groups and orally administered the corresponding formulation (*n* = 4). At 24 h post administration, the small intestine and lung tissues of surviving mice in each group were collected, rinsed with ice-cold PBS. One portion of the tissues was used for HE staining, while the other portion was homogenized. Total RNA was then extracted from the homogenized tissues using the Trizol method, reverse-transcribed into cDNA, and quantified for adenoviral genomic copy number by qPCR to evaluate viral retention in the tissues.

### 2.28. Examination of H&E-Stained Tissue Sections

The collected mouse tissue samples were promptly placed in pre-cooled 4% paraformaldehyde (PFA) in phosphate-buffered saline (PBS, pH 7.4) and fixed at 4 °C for 24 h. Following fixation, the tissues were dehydrated through a graded ethanol series, cleared in xylene, embedded in paraffin, and sectioned at a thickness of 4–5 μm. The sections were then deparaffinized, rehydrated, and stained with hematoxylin and eosin (H&E). After staining, the sections were dehydrated, cleared, and mounted with neutral balsam. The stained sections were observed under a light microscope (bright-field) to examine the tissue morphological structure. Digital images were captured using a microscope-mounted camera.

### 2.29. Statistical Analysis

Statistical analysis of the experimental data was completed by Graphpad Prism 9.0 software. All results were generated based on three independent replicated experiments and presented as mean ± standard deviation (SD). Statistical analyses were performed using one-way ANOVA or two-way ANOVA. Tukey’s HSD test was applied following one-way ANOVA to compare all pairwise groups. Statistical significance was determined when *p* < 0.05.

## 3. Results

### 3.1. Screening of Low-Allergenic Pollen

**Figure 1:**
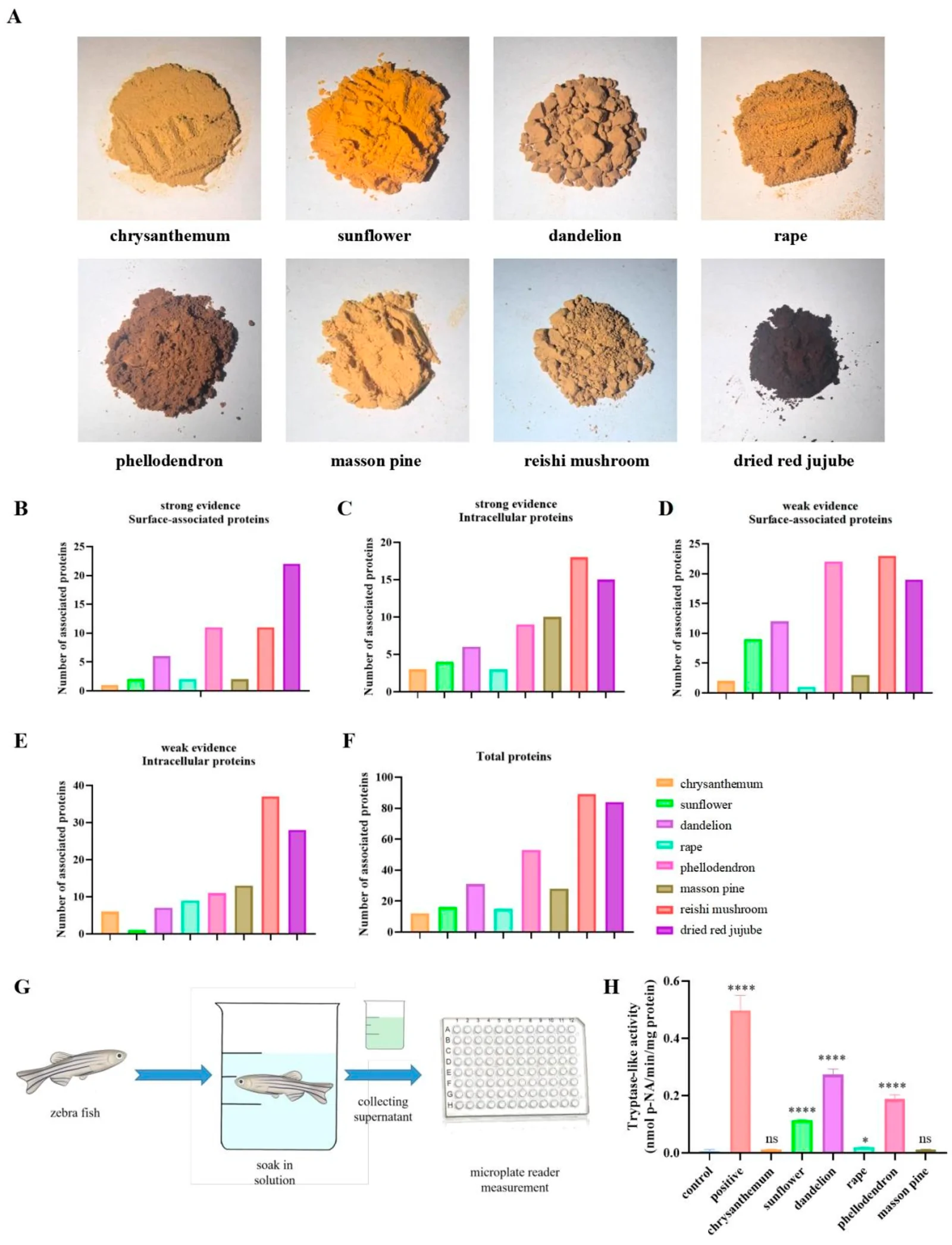
Selection of Low-Allergenic Pollen. (**A**) Representative images of eight types of pollen. Proteomic analysis showing: (**B**) Number of strongly allergenic proteins in pollen surface-associated proteins, (**C**) Number of strongly allergenic proteins in pollen internal proteins, (**D**) Number of weakly allergenic proteins in pollen surface-associated proteins, (**E**) Number of weakly allergenic proteins in pollen internal proteins, (**F**) Total number of allergenic proteins in pollen. (**G**) Schematic diagram of the zebrafish allergy experiment. (**H**) Tryptase-like activity in zebrafish larvae following exposure to various pollen extracts. (*n* = 6, ****: *p* < 0.0001; *: *p* < 0.05; ns: not significant, *p* > 0.05).

As the male gametophyte of plants, natural pollen, with its inherent micron-sized particle morphology, diverse surface topography, and unique biocompatibility, has long been regarded as an ideal template or raw material for constructing drug delivery systems. However, its allergenicity is the primary challenge to be overcome. Not all pollens possess the same degree of sensitization potential. Therefore, selecting and developing a novel carrier material that combines the natural structural advantages of pollen with low biological risk is key to overcoming the bottlenecks in this field. Figure 1A shows the physical samples of chrysanthemum (*Chrysanthemum indicum* L.), sunflower (*Helianthus annuus* L.), dandelion (*Taraxacum officinale* F.H. Wigg.), rape (*Brassica napus* L.), phellodendron (*Phellodendron amurense* Rupr.), masson pine (*Pinus massoniana* Lamb.), reishi mushroom spore (*Ganoderma lucidum* (*Curtis*) P. Karst.), and dried red jujube (*Ziziphus jujuba* Mill.) pollen.

Based on proteomics data, this study conducted a systematic quantitative analysis of allergen proteins in the eight pollen types. Based on their homology to known allergens and the strength of evidence, proteins were classified as strongly allergen-associated or weakly allergen-associated. They were further distinguished by their localization as surface or internal proteins (Figure 1B–F). Among the eight pollens, chrysanthemum and rape pollen consistently showed the lowest number of allergen proteins across all categories, particularly for strongly allergen proteins located on the surface. Sunflower and masson pine pollens had similar total amounts of allergen proteins, but the distribution of these proteins revealed a critical difference. Sunflower pollen had a higher number of surface-associated proteins compared to masson pine pollen. This implies that sunflower antigens are more likely to interact directly with immune cells in the mucosa, posing a higher risk of eliciting a type I hypersensitivity reaction. In contrast, the allergenic proteins in masson pine pollen were primarily concentrated internally. Its sensitization process relies on the physical rupture of pollen grains, which may, to some extent, reduce its immediate sensitizing potency under natural exposure conditions.

The total amount of allergen proteins was further increased in dandelion pollen and phellodendron pollen. Both exhibited a pattern dominated by internal proteins. However, phellodendron pollen was relatively prominent in the abundance of strongly allergen proteins on its surface, suggesting potentially stronger direct sensitizing capacity. Finally, reishi mushroom spore and dried red jujube pollen were excluded from further comparative analysis due to the higher absolute numbers of both surface and internal proteins compared to other samples.

Synthesizing the above analyses, this study constructed a preliminary proteomics-based risk assessment framework for sensitization: Chrysanthemum and rape pollen posed the lowest risk; masson pine pollen, due to the “hidden” nature of its main antigens, was of potential risk; sunflower pollen showed a clearly elevated risk due to its richness in surface antigens; dandelion and Phellodendron pollens constituted categories with higher potential sensitization. This proteomic analysis systematically elucidated the composition and distribution characteristics of allergen proteins in different pollens from both qualitative and quantitative perspectives, providing important molecular-level theoretical predictions for assessing their potential sensitization risk. However, protein abundance and localization do not completely equate to their actual sensitizing capacity. Their immunogenicity, epitope exposure, and interaction with the host immune system need to be ultimately confirmed in a physiological in vivo environment. Therefore, to functionally validate the above predictions and to clarify how these differences translate into actual allergic response intensity in a holistic biological system, this study further conducted a systematic in vivo allergenicity assessment of the chrysanthemum, sunflower, dandelion, rape, phellodendron, masson pine pollens mentioned above through animal experiments.

This study systematically evaluated the sensitization potential of different pollens using a zebrafish allergy model. The intensity of the allergic reaction was quantitatively reflected by measuring the absorbance of the supernatant at 405 nm after co-incubation of zebrafish with the substrate. The experimental procedure is shown in Figure 1G. The absorbance at 405 nm (OD_405_) was plotted against p-NA concentration (nmol/mL). The linear regression equation was y = 0.0086x + 0.0147, with a correlation coefficient R^2^ = 0.9990, indicating excellent linearity within the measured range (0–100 nmol/mL). The results showed that the OD value of the negative control group remained at a low level, while the positive control group exhibited a strong response with a relative activity of approximately 0.5 nmol p-NA/min/mg protein, indicating that the model was successfully established. Among the tested pollens, the chrysanthemum and rape flower pollen treatment groups showed enzyme activity levels similar to the negative control, both around 0.1 nmol p-NA/min/mg protein, showing no significant difference, indicating extremely low allergenicity. The masson pine pollen group, although slightly higher than the negative control, was still significantly lower than the positive control, suggesting it has only a mild sensitization potential. In contrast, dandelion, sunflower, and Phellodendron pollens showed significant differences compared to the negative control, demonstrating that these three pollens all triggered a strong allergic reaction (as indicated by elevated tryptase-like activity) in the zebrafish model (Figure 1H). Based on these results, chrysanthemum, rape, and masson pine pollens, identified as materials with low sensitization potential, were selected for subsequent in-depth mechanistic studies on material properties.

### 3.2. Preparation and Characterization of Sporopollenin

**Figure 2:**
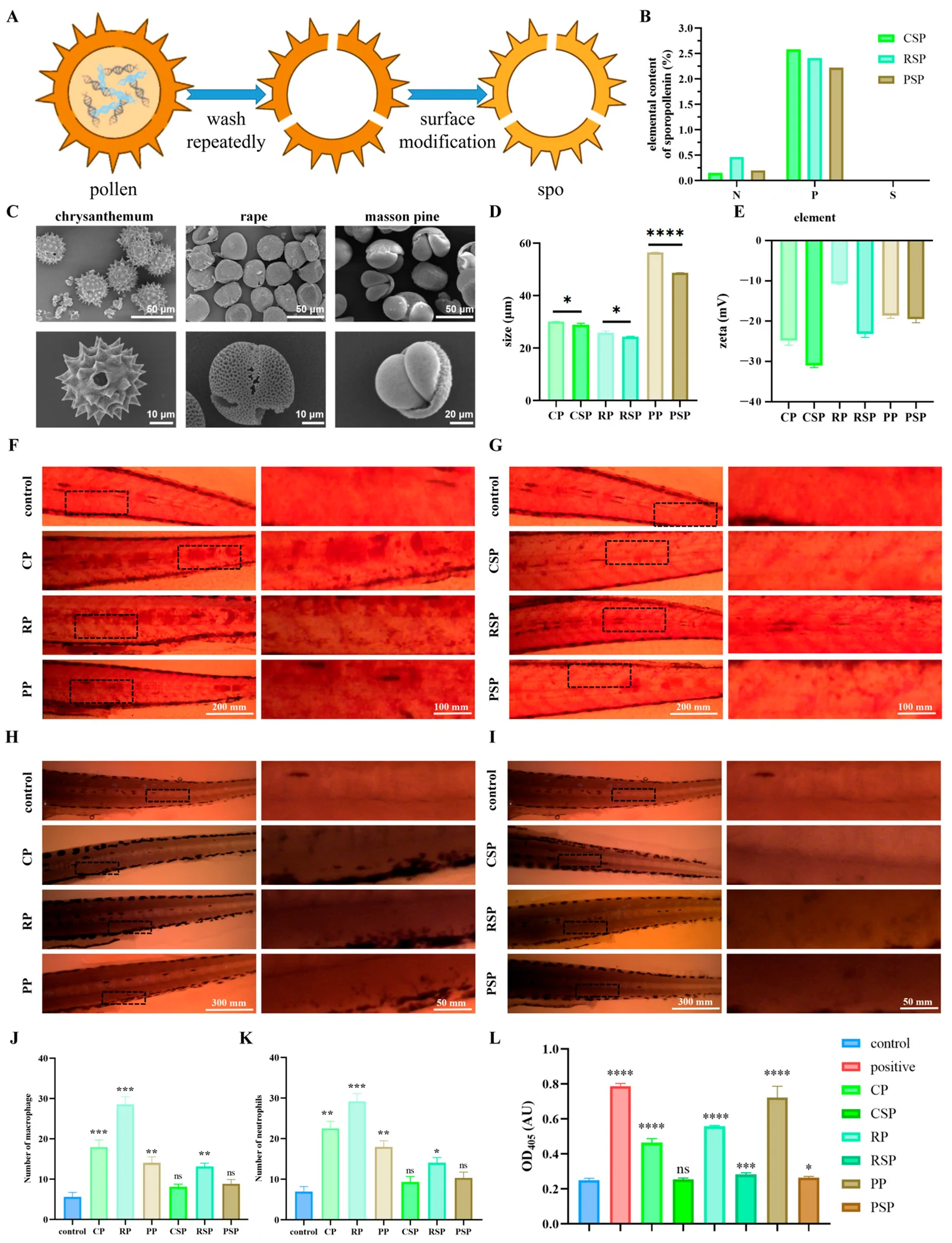
Preparation and Characterization of Sporopollenin. (**A**) Schematic diagram of the preparation process of sporopollenin. (**B**) Elemental analysis of three types of sporopollenin. (**C**) The sporopollenin of chrysanthemum, rape flower, and masson pine pollen was characterized under a scanning electron microscope. Analysis of (**D**) particle size and (**E**) Zeta potential of pollen and sporopollenin from chrysanthemum, rape flower, and masson pine pollen. (**F**) Effect of incubation with different types of pollen on macrophage count in zebrafish. (**G**) Effect of incubation with different types of sporopollenin on macrophage count in zebrafish. The dashed boxes in (**F**,**G**) indicate the regions that are magnified on the right side. (**H**) Effect of incubation with different types of pollen on neutrophil count in zebrafish. (**I**) Effect of incubation with different types of sporopollenin on neutrophil count in zebrafish. (**J**) Number of macrophages recruited to the zebrafish tail. (**K**) Number of neutrophils recruited to the zebrafish tail. (**L**) Allergy tests on zebrafish conducted at higher concentrations to examine allergic reactions to pollen and sporopollenin from chrysanthemum, rape flower, and masson pine pollen. (*n* = 15, ****: *p* < 0.0001; ***: *p* < 0.001; **: *p* < 0.01; *: *p* < 0.05; ns: not significant, *p* > 0.05).

Sporopollenin was prepared from the three pollens selected in the previous experiments. The preparation process is shown in Figure 2A. To evaluate the removal of N, P, and S-containing components, the contents of nitrogen, phosphorus, and sulfur in the prepared sporopollenin were determined. The protein content was calculated based on the elemental analysis results (Figure 2B). The protein content, derived by multiplying the nitrogen content by a factor of 6.25, showed that the protein content of all three sporopollenin types was no greater than 4%. The protein removal efficiency exceeded 90%, demonstrating successful sporopollenin preparation with equally high efficiency for all three pollens, indicating the broad applicability of this method.

Morphological characterization by scanning electron microscopy (SEM) is shown in Figure 2C. The prominent spike structures on the surface of chrysanthemum sporopollenin significantly increase its specific surface area. This not only effectively enhances the surface charge density, leading to a more negative zeta potential and improved stability, but also leaves the main structure largely unchanged. Chrysanthemum sporopollenin possesses a relatively intact hollow cavity structure with only three germination pores connecting to the exterior, providing a robust framework for subsequent drug loading. Rape sporopollenin exhibits a regular spherical outline with deep surface grooves and three symmetrical germination slits. Its drug-loading performance is likely inferior to that of chrysanthemum sporopollenin. Masson pine sporopollenin features a unique double-sac structure. Its internal cavity is limited, with a greater volume occupied by the air sacs, which are sealed compartments not connected to the outside, thus offering little benefit for subsequent drug loading.

Particle size analysis indicated that the size of sporopollenin from all three pollens decreased compared to the original pollens. The particle size of chrysanthemum sporopollenin decreased from 30.26 μm to 28.69 μm, while the particle size of rape flowers sporopollenin decreased from 26.86 μm to 23.75 μm. The reduction in particle size for both was relatively small, but the particle size of pine pollen sporopollenin showed the most significant decrease, dropping from 53.49 μm to 49.67 μm. The reduction was relatively smaller for chrysanthemum and rape sporopollenin, while masson pine sporopollenin showed the most significant decrease, dropping substantially from approximately 60 µm. This suggests that the exine structure and composition of different pollens are affected to varying degrees during the removal of intracellular materials and non-sporopollenin components. The air sac structure of masson pine pollen, in particular, may be significantly impacted during processing, leading to a greater change in particle size for masson pine sporopollenin (Figure 2D). Further analysis from an electrochemical stability perspective revealed that zeta potential results consistently showed sporopollenin to have a lower surface charge. This indicates that the preparation process effectively enhances interparticle electrostatic repulsion, thereby improving dispersion stability. Chrysanthemum sporopollenin exhibited the lowest potential (−31.6 mV), demonstrating the strongest potential for electrostatic stabilization. Rape sporopollenin had a relatively higher potential (−24.3 mV), suggesting that its surface groove characteristics might lead to lower repulsive forces, making it prone to aggregation. Masson pine pollen and its sporopollenin showed minimal change in potential. Due to its anemophilous nature—being lighter with air sacs—and the fact that the sporopollenin extraction process does not damage the cavity structure, its stability changed little (Figure 2E). In summary, sporopollenin preparation not only significantly altered the physical size of the particles but, more importantly, influenced their colloidal behavior by modulating surface charge. Chrysanthemum sporopollenin, while maintaining a relatively small change in particle size, achieved a significant decrease in potential, exhibiting superior stability regulation characteristics.

Macrophages play a crucial role in immune responses. Analysis of staining results showed no observable macrophage aggregation in the tail region of the negative control group. Significant recruitment of macrophages was observed in the tails of zebrafish in all other groups, indicating that pollen components can induce marked macrophage recruitment. The pollen groups (Figure 2F) showed a significant difference in macrophage recruitment compared to the sporopollenin groups (Figure 2G). The ranking for inflammatory cell count and allergenicity was: rape pollen > chrysanthemum pollen > masson pine pollen > rape sporopollenin > masson pine sporopollenin > chrysanthemum sporopollenin. Notably, masson pine sporopollenin and chrysanthemum sporopollenin showed no significant difference compared to the negative control group, demonstrating that sporopollenin, with proteins removed, exhibits reduced allergenicity (Figure 2J). Similarly, Sudan Black staining was used to detect the presence of neutrophils. Figure 2H,I shows that no obvious neutrophil aggregation was observed in the tail region of the negative control group. Other groups showed varying degrees of strong neutrophil aggregation in the tail, indicating that pollen components induce an inflammatory response leading to neutrophil recruitment. The differential aggregation levels can be used to judge the relative allergenicity strength of different pollen components. Statistical analysis of neutrophil recruitment counts in zebrafish tails (Figure 2K) revealed a normal level in the negative control. The pollen groups significantly increased neutrophil counts, while the sporopollenin groups caused a slight increase. The ability to induce neutrophil recruitment ranked as: rape pollen (RP) > chrysanthemum pollen (CP) > masson pine pollen (PP) > rape sporopollenin (RSP) > masson pine sporopollenin (PSP) > chrysanthemum sporopollenin (CSP). Masson pine sporopollenin and chrysanthemum sporopollenin again showed no significant difference from the negative control, further proving the lower allergenicity of deproteinized sporopollenin, with chrysanthemum sporopollenin exhibiting the optimal low-allergenicity properties, probably contributed from its optimal combination of small particle size (enhanced cellular uptake).

This study further conducted allergic tryptase detection in adult zebrafish. The results are shown in Figure 2L. Compared to the original pollens, the sporopollenin extracts from all three pollens showed lower OD values at high concentrations. This indicates that the sensitization reaction is primarily associated with water-soluble or protein components in pollen, while sporopollenin itself is not the main allergen. Among the sporopollenin groups, the chrysanthemum sporopollenin group had a relatively low OD value. Although slightly higher than the negative control, the difference was not significant, suggesting its non-sensitizing potential even at high concentrations. In contrast, the sporopollenin groups from rape and masson pine pollens, while also showing low OD values, exhibited a statistically significant difference compared to the negative control group.

### 3.3. pH-Responsive Properties of Sporopollenin

**Figure 3:**
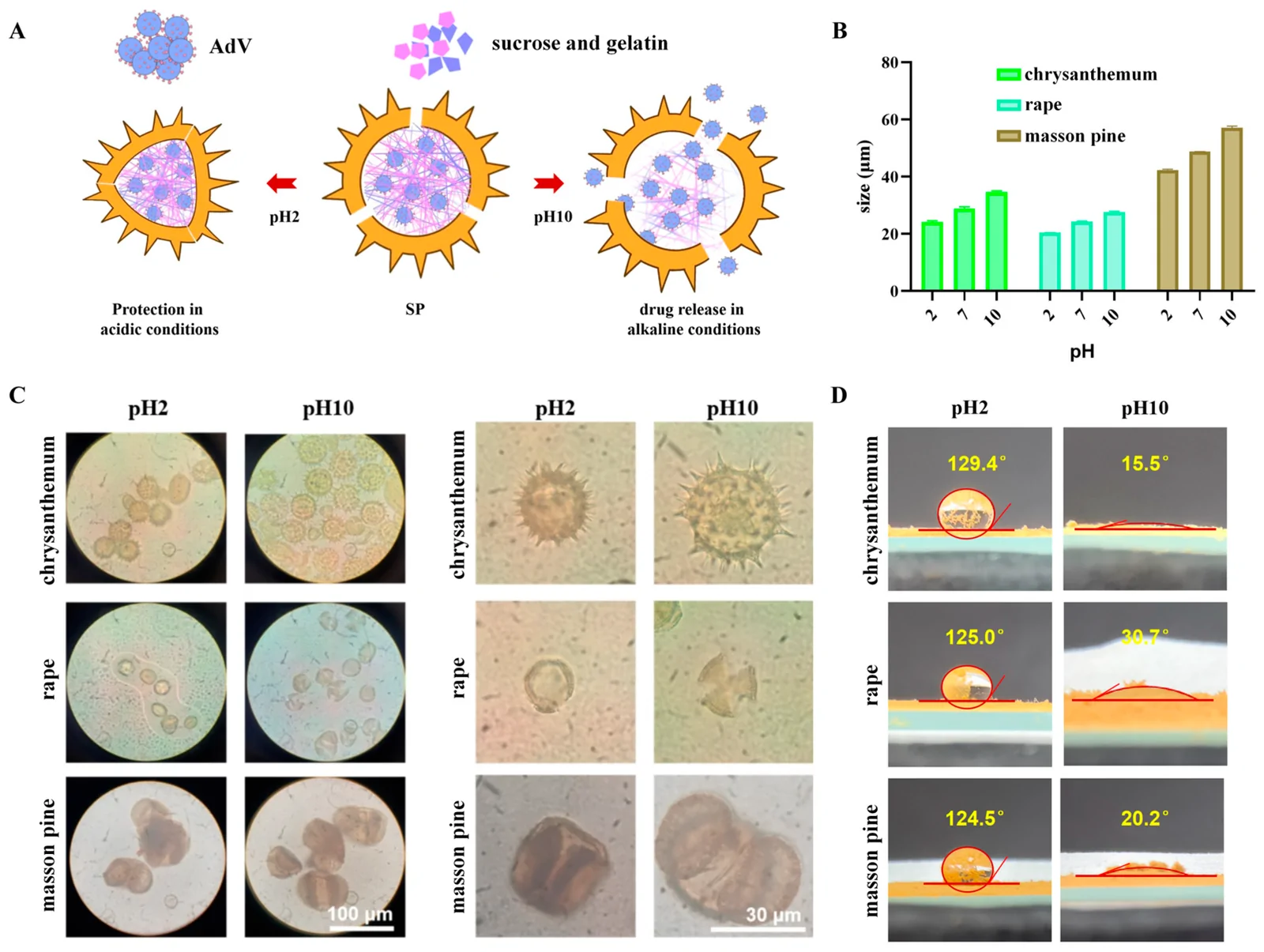
pH-responsive Analysis of Sporopollenin. (**A**) Schematic diagram of sporopollenin deformation under acidic and alkaline conditions. (**B**) The particle size variations in the spore of chrysanthemum, rape flower and pollen masson pine within the pH range of 2 to 10 (*n* = 3). (**C**) Observation of the microscopic morphology of three spore proteins under different pH conditions. (**D**) The contact angles of three spores at different pH values.

The surface chemical properties of sporopollenin exhibit a specific pH response. In our previous study, we found that under acidic conditions, functional groups like carboxyl groups on the sporopollenin exine become protonated, rendering the surface electrically neutral or positively charged. Concurrently, the molecular conformation may contract, which is unfavorable for molecular adsorption and diffusion. As the pH increases, these functional groups gradually deprotonate, increasing the negative surface charge of the carrier. This enhances electrostatic attraction with positively charged drug molecules [37]. Simultaneously, the germination pores of the sporopollenin open, and the internal cavity volume increases, facilitating the diffusion of substances (Figure 3A).

To verify the pH responsiveness of the sporopollenin carriers, the dynamic changes in the hydrodynamic size of the three sporopollenin types were systematically measured across a pH range of 2.0 to 10.0. The results revealed pH dependence in the size of all three sporopollenin types, with a universal trend: the particle size increased as the environmental pH transitioned from acidic to alkaline (Figure 3B).

Microscopic morphological observations of the three sporopollenin types under different pH conditions further confirmed the generally observed phenomenon of “acid-induced shrinking and alkali-induced swelling.” In the acidic environment of pH 2.0, all three sporopollenin types exhibited a contracted structure and a strongly hydrophobic surface. When the environment shifted to pH 10.0, all samples underwent varying degrees of volume expansion, creating more favorable pore channels and surface area for the diffusion and adsorption of drug molecules. However, significant differences existed in the structural responsiveness of the different sporopollenin types, which profoundly impacted their performance as carriers. During alkaline swelling, the characteristic spike structures of chrysanthemum sporopollenin remained intact, with no obvious cracks or pores appearing on the surface, indicating high mechanical strength and structural stability of its exine. This uniform and controllable swelling pattern is likely beneficial for forming a stable and reproducible drug-loading environment. In contrast, rape sporopollenin exhibited a more drastic morphological change under alkaline conditions: its three germination slits completely opened, and obvious cracks appeared on the exine. Such structural damage could compromise the integrity of the carrier, would be predicted to cause a burst release of the loaded drug during subsequent processing or delivery, thereby introducing uncertainty in loading stability and release behavior. The structural response of masson pine sporopollenin was the most unique: under acidic conditions, its characteristic air sac structure underwent significant inward contraction, compressing the internal space. This excessive deformation may not only affect the initial drug loading capacity but could also interfere with the controlled release kinetics of the drug during environmental changes in vivo due to secondary expansion or contraction of the air sacs, leading to unpredictable release behavior (Figure 3C).

Surface wettability analysis, shown in Figure 3D, revealed that the contact angles of all three sporopollenin types were highly sensitive to pH changes, following a remarkably consistent trend. Under the acidic condition of pH 2.0, the contact angles for chrysanthemum, rape, and masson pine sporopollenin were as high as 129.4°, 125.0°, and 124.5°, respectively, exhibiting typical hydrophobic characteristics. When the medium pH increased to 10.0, the contact angles sharply decreased to 15.5°, 30.7°, and 20.2°, indicating a transformation to a highly hydrophilic state. This drastic wettability switch was universally observed across the three sporopollenin types from different sources, strongly proving that their surface chemical properties are governed by pH.

### 3.4. Mucosal Retention of Sporopollenin In Vitro and In Vivo

**Figure 4:**
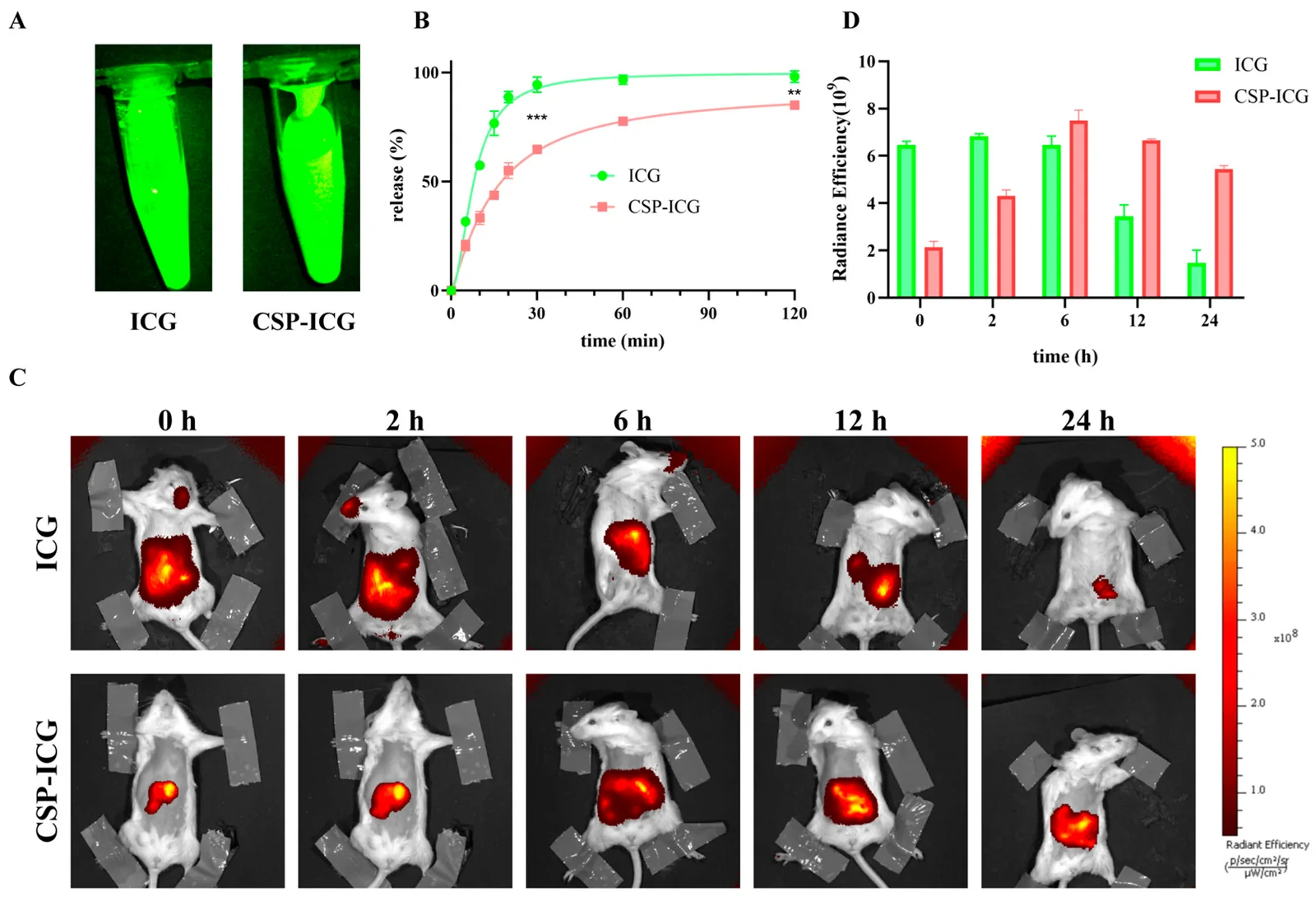
In vivo and in vitro Mucoadhesion Experiments. (**A**) Schematic diagram of the fluorescence intensity of ICG and CSP-ICG. (**B**) The in vitro experiment of simulating the retention property of the mucosa using the sporopollenin carrier (*n* = 3). (**C**) Dynamic images of gastrointestinal tract retention in mice via in vivo imaging for ICG and CSP-ICG. The color scale range from 5 × 10^7^ to 5 × 10^8^. (**D**) Quantitative analysis of fluorescence intensity from the in vivo imaging. (***: *p* < 0.001; **: *p* < 0.01).

The unique physical structure of chrysanthemum pollen facilitates its retention on mucosal surfaces. According to Figure 4B, under the simulated flushing conditions, the release amount of ICG solution reached 85.3% within 0.5 h. This indicates weak adhesion of the free drug on the mucosal surface, making it susceptible to rapid clearance by body fluids or mucus and difficult to maintain an effective local concentration over an extended period. In contrast, the release percentage of ICG loaded within sporopollenin was consistently and lower than that of the free ICG solution throughout the 2 h monitoring period, with a relatively flat release profile, the release curve was relatively gentle—the release amount at 30 min was only 54.8%, and the cumulative release amount after 2 h was 78.5%, indicating that some of the drug remained on the simulated mucosa. This clearly proves that the sporopollenin carrier effectively encapsulated and anchored ICG on or within the simulated mucosal surface/structure, significantly slowing the rate of drug clearance by flushing and enabling prolonged, slow release.

This study further investigated in vivo mucosal retention. Firstly, an ICG solution and a sporopollenin-loaded ICG solution with equal fluorescence intensity were prepared (Figure 4A). Comparing the gastrointestinal retention results of the two solutions in mice (Figure 4C), the fluorescence intensity of chrysanthemum sporopollenin was lower than that of the solution group prior to 6 h, but by the 6 h mark, its fluorescence intensity had surpassed that of the solution group and exhibited a broader distribution range. The fluorescent signal persisted and gradually released over 24 h, indicating a stable gastrointestinal retention effect. In contrast, the solution group demonstrated stronger fluorescence intensity and a wider distribution within the first 6 h, but most of the fluorescence diminished after 12 h, leaving only a weak fluorescent signal by 24 h (Figure 4D). This demonstrates that chrysanthemum sporopollenin effectively increased the retention time of the drug in the gastrointestinal tract.

### 3.5. Optimization and Characterization of Drug Loading Performance

**Figure 5:**
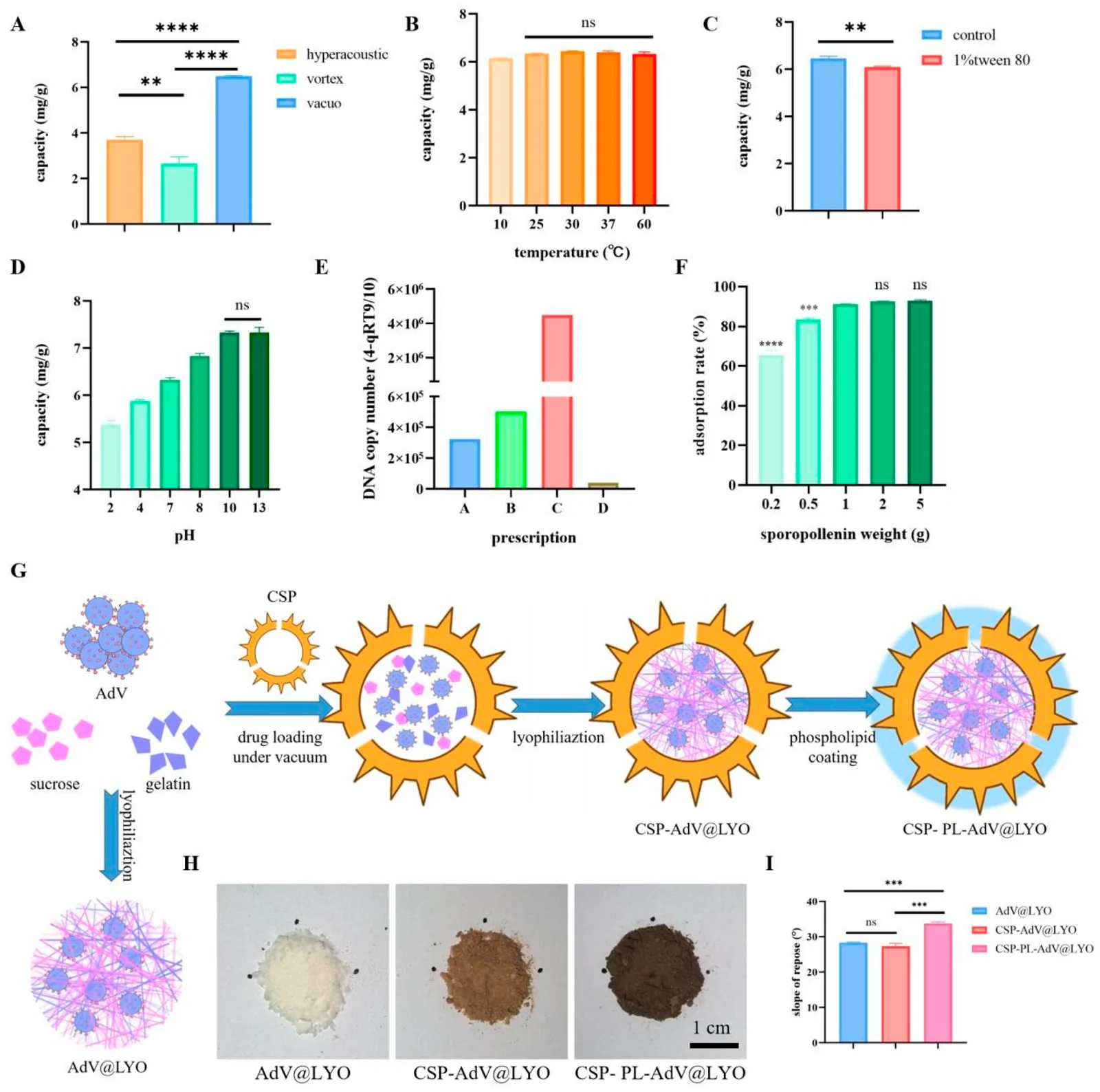
Optimization of Drug Loading Properties and Preparation Characterization of Chrysanthemum Sporopollenin. (**A**) Drug delivery performance of CSP under three drug loading methods: ultrasound, vortex, and vacuum. (**B**) Effect of temperature on the drug delivery performance of CSP carriers. (**C**) Effect of surfactants on the drug delivery performance of CSP carriers. (**D**) Effect of environmental pH on the drug delivery performance of CSP carriers. (**E**) The number of viral DNA copies released after the administration of prescriptions A, B, C, and D. (**F**) Study on the dosage of CSP. (**G**) Schematic diagram of the preparation processes for three drug loading systems. (**H**) Photographs of the three drug loading systems. (**I**) Comparison of the angle of repose for the three drug loading systems. (****: *p* < 0.0001; ***: *p* < 0.001; **: *p* < 0.01; ns: not significant, *p* > 0.05).

To facilitate subsequent drug loading, this study optimized the drug loading methods and formulations. First, the drug loading performance of chrysanthemum sporopollenin under three methods—ultrasonication, vortexing, and vacuum—was systematically evaluated. The results (Figure 5A) showed that chrysanthemum sporopollenin achieved the highest drug loading capacity with vacuum loading, significantly outperforming ultrasonication and vortexing. This indicates that vacuum negative pressure may effectively remove air from its porous structure, enhancing the contact efficiency at the drug-carrier interface. The results suggest that the vacuum loading method can maximize the efficacy of the drug loading system. The effect of temperature on the carrier’s loading performance was further investigated (Figure 5B). The drug loading capacity of chrysanthemum sporopollenin showed little fluctuation between 10 and 60 °C, suggesting it is not significantly affected by temperature changes. Next, the effect of surfactant additives was examined (Figure 5C). Tween 80, a non-ionic surfactant, can reduces the interfacial tension between different surface and the aqueous medium, thereby enhancing wetting and facilitating the displacement of weakly bound ions or proteins. Contrary to conventional expectations, the addition of 1% Tween 80 did not promote drug loading but instead exerted a significantly inhibitory effect on sporopollenin. This phenomenon may stem from the unique surface properties of sporopollenin: as an entomophilous pollen, chrysanthemum sporopollenin has a relatively regular, lipophilic sculptured layer on its exine. Tween 80 molecules may form a competitive adsorption layer on its surface, slightly interfering with drug loading. This study then measured the variation in the drug loading capacity of chrysanthemum sporopollenin across a pH range of 2.0 to 13.0. The results (Figure 5D) showed that the drug loading capacity increased with rising pH and stabilized after pH > 10.0. This aligns with the previously observed pH responsiveness of sporopollenin, proving that the increased volume of sporopollenin with pH correlates with enhanced drug loading capacity.

Subsequently, this study compared the effects of different freeze-drying protectant compositions on the lyophilized product. Among them, Formulations B, C, and D all formed structurally loose, intact lyophilized cakes with a moisture content below 3%, demonstrating good physical stability. Further qPCR analysis using one pair of specific primers to detect the viral genome copy number after lyophilization showed (Figure 5E) that Formulation C exhibited the highest viral DNA copy number, approximately an order of magnitude higher than other formulation groups. This indicates a significant advantage of this formulation in maintaining viral structural integrity. Sucrose, as a non-reducing disaccharide, can maintain the spatial integrity of the viral structure by forming a glassy state and replacing water molecules binding to the viral protein surface. Additionally, the inclusion of gelatin constructs a robust porous framework, significantly improving the physical structure of the lyophilized cake and its reconstitution properties. In summary, based on a comprehensive evaluation of three key indicators—lyophilized appearance, moisture content, and viral genome copy number retention rate—Formulation C was determined to be the optimal freeze-drying protectant formula for adenovirus in this experiment. The dosage of sporopollenin was then investigated (Figure 5F). When the sporopollenin dosage was 0.2 g, the adsorption rate was approximately 60%; increasing to 0.5 g raised the adsorption rate to about 83%; at 1 g, the adsorption rate could exceed 90%. Further increasing the dosage to 2 g and 5 g did not significantly improve the adsorption rate, indicating that adsorption had essentially reached equilibrium at 1 g, meaning the available adsorption sites on the carrier surface were approaching saturation.

Figure 5G shows the preparation flowchart for the three samples. From the physical images of the lyophilized powders, AdV@LYO appears as a white powder, while CSP-AdV@LYO and CSP-PL-AdV@LYO lyophilized powders are brownish in color (Figure 5H). According to the flowability assessment, the angle of repose for the AdV@LYO sample was about 28°, and slightly lower for CSP-AdV@LYO at about 27°. However, both samples had an angle of repose less than 30°, indicating strong particle flowability. The angle of repose for CSP-PL-AdV@LYO increased significantly to about 35°, markedly higher than the other two groups (Figure 5I). The angle of repose is a key physical parameter for evaluating powder flowability; a larger angle indicates greater interparticle adhesion or friction, resulting in poorer flowability. In this experiment, CSP-AdV@LYO showed slightly better flowability than AdV@LYO, possibly because sporopollenin, as a microcarrier, provides a more regular particle morphology. The significantly reduced flowability of CSP-PL-AdV@LYO is primarily attributed to the additional surface stickiness introduced by the phospholipid coating layer. The phospholipid forms a thin film on the particle surface, likely enhancing interparticle cohesion through intermolecular forces, thereby increasing flow resistance.

### 3.6. In Vitro Stability of Drug-Loaded Sporopollenin

**Figure 6:**
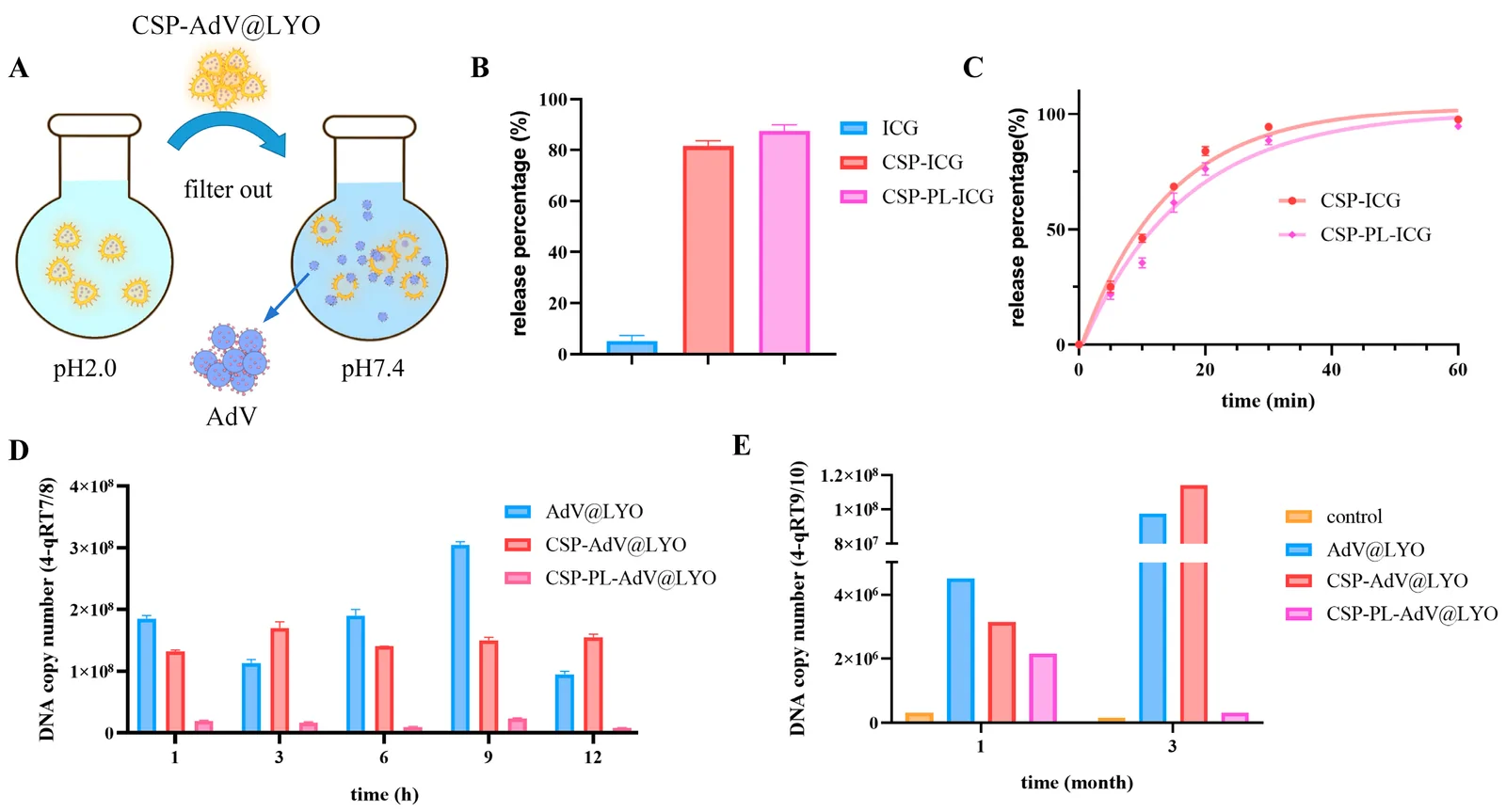
In vitro and in vivo Stability of Drug-loaded Chrysanthemum Sporopollenin. (**A**) Schematic diagram of the in vitro stability experiment procedure. (**B**) In vitro release assay of ICG, CSP-ICG, and CSP-PL-ICG under simulated gastrointestinal tract conditions. (**C**) Release curve of ICG-loaded sporopollenin in simulated intestinal fluid. (**D**) The release amounts of DNA after 1, 3, 6, 9, and 12 h of release in the simulated intestinal fluid for AdV@LYO, CSP-AdV@LYO, and CSP-PL-AdV@LYO were examined. (**E**) Comparison of viral copy numbers of the virus stock solution, AdV@LYO, and CSP-AdV@LYO at 1 and 3 months during the long-term test.

This study focused on investigating the protective effect of sporopollenin on the encapsulated drug, particularly examining drug stability under simulated pH changes encountered after oral administration (Figure 6A). First, the acid release of sporopollenin loaded with ICG was examined. As shown in Figure 6B, because the molecular structure of ICG rapidly degrades in acidic solutions, generating non-fluorescent, colorless products, the concentration of unprotected ICG solution did not exceed 10% of the original concentration, indicating fluorescence quenching in the acidic medium. In contrast, ICG protected by sporopollenin was shielded in the acid and subsequently released under alkaline conditions, achieving release degrees of 83% and 89%, respectively. The sporopollenin coated with an outer phospholipid layer offered slightly better protection, although the difference was not statistically significant. In the simulated intestinal fluid release experiment (Figure 6C), CSP-ICG and CSP-PL-ICG exhibited similar release profiles, demonstrating that the outer phospholipid coating did not affect the release process.

As shown in Figure 6D, AdV@LYO showed a relatively high release amount as early as 1 h, indicating a faster initial release, which may be related to its carrier structure or coating properties. Its release peaked at 9 h, significantly higher than the other two formulations, suggesting optimal cumulative release efficiency at that time point. The release amount of CSP-AdV@LYO was at a moderate level at all time points, with a relatively flat release curve and no significant peak, indicating a potentially more gradual or sustained release behavior. CSP-PL-AdV@LYO exhibited the lowest release amount at all time points, indicating poor release efficiency, likely due to carrier adsorption or excessive coating restricting release. In comparison, CSP-AdV@LYO demonstrated the most stable and sustained release kinetics. Its release amount changed gently throughout the observation window (1 to 12 h) without drastic fluctuations, suggesting a more controllable and stable release behavior. Although the release amount of AdV@LYO exceeded that of CSP-AdV@LYO, its entire release process was unstable, especially with a sudden surge at 9 h, posing a potential safety risk. The detection results confirmed the above release trends, enhancing data reliability. From the perspective of controlling release behavior consistency and predictability, the stable, sustained release characteristics exhibited by CSP-AdV@LYO may be more conducive to achieving steady drug release and delivery in the intestine, offering potential advantages in drug delivery systems requiring stable blood concentration or local action.

To confirm its long-term stability, the long-term stability test was conducted. Throughout the long-term testing period, all three lyophilized samples exhibited relatively stable virus release capability. The ratio of their DNA copy number to that of the original virus stock remained largely consistent across time points, preliminarily indicating that the lyophilization process provided a certain degree of protection for viral activity (Figure 6E). Notably, after 3 months, the release profiles of the AdV@LYO and CSP-AdV@LYO groups showed a more pronounced upward trend, suggesting that viral activity remained more stable, or may have even been preserved, under long-term conditions. This could be attributed to the physical encapsulation and protective effect of the sporopollenin carrier, slowing the activity decay of the virus during storage. In contrast, the CSP-PL-AdV@LYO group, with the phospholipid-coated sporopollenin carrier, exhibited poorer release during the same period. This result indicates that sporopollenin, as a microcarrier, holds an advantage in maintaining the long-term stability of adenovirus. Its porous structure likely provides physical isolation and protection for the virus, thereby better preserving viral structural integrity and release activity under long-term conditions. In summary, CSP-AdV@LYO, prepared via the lyophilization process combined with the sporopollenin carrier, demonstrated superior viral genome copy number retention rate in the long-term stability test. These results suggest that CSP-AdV@LYO may possess favorable long-term storage stability, although extended testing under ICH-guided conditions is required to establish a definitive shelf-life.

### 3.7. In Vivo Performance and Safety Evaluation of Drug-Loaded Sporopollenin

**Figure 7:**
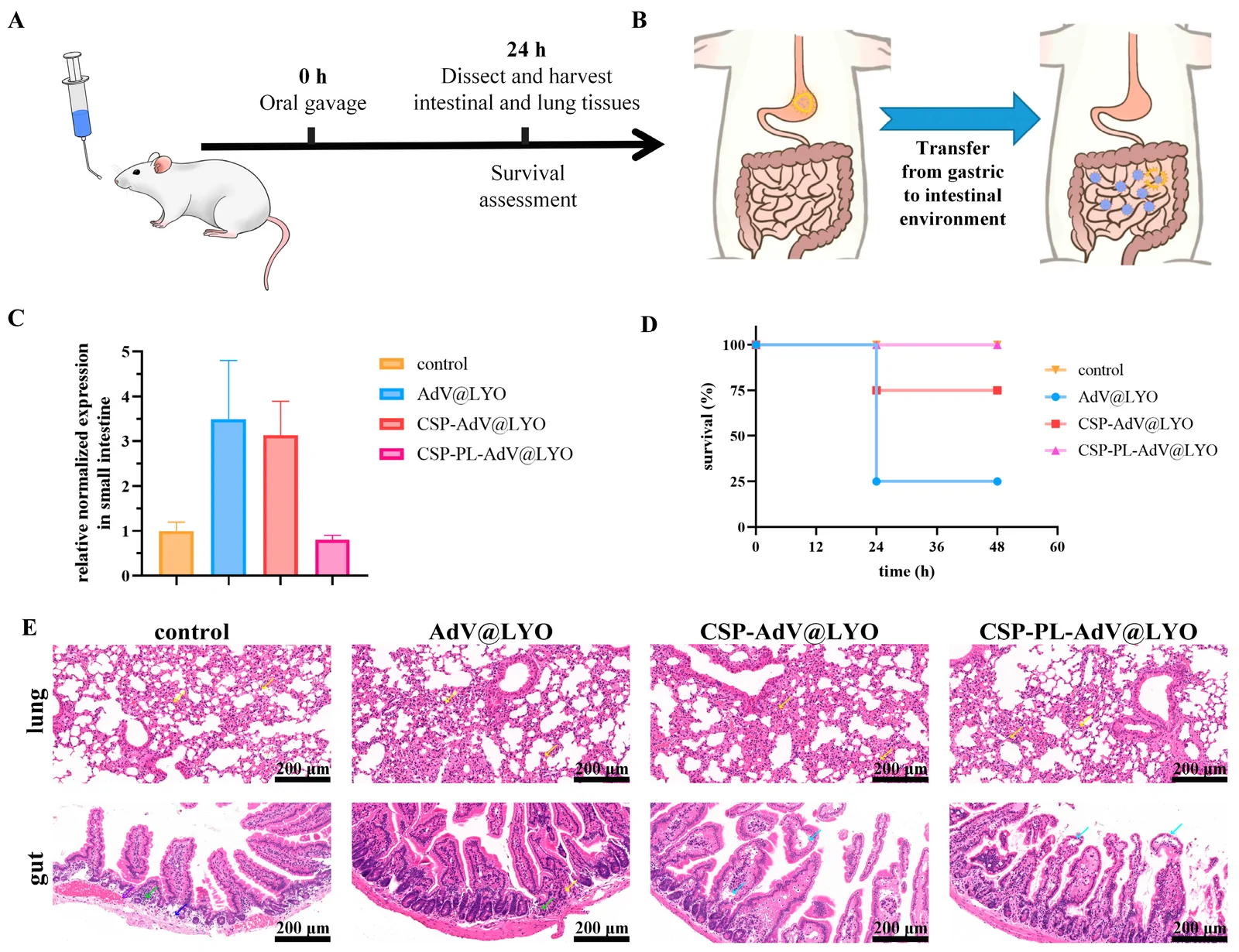
In vivo Stability and Safety Evaluation of Drug-loaded Sporopollenin. (**A**) Schematic diagram of the animal experimental procedure. (**B**) Schematic diagram of drug release and colonization after the formulation travels from the stomach to the intestinal tract in mice. Viral colonization in the (**C**) intestines (*n* = 4) and (**D**) survival rate assessment (*n* = 4). (**E**) H&E staining images of tissue sections from the lungs and intestines of mice. In the lung sections, yellow arrows indicate granulocyte infiltration in the alveolar walls. In the intestinal sections, blue arrows indicate loosely arranged intestinal glands in the lamina propria; yellow arrows indicate mild hyperplasia and replacement of connective tissue; green arrows indicate lymphocytic infiltration with small focal aggregations; cyan arrows indicate separation of the mucosal epithelium from the lamina propria; and purple arrows indicate vascular congestion.

Previous experiments demonstrated the excellent in vitro stability of drug-loaded sporopollenin. This study further evaluated its in vivo stability. The experimental procedure is shown in Figure 7A, focusing on the release and colonization of the virus in the intestinal tract following oral administration and passage through the mouse stomach (Figure 7B). The results (Figure 7C) showed that compared to the wild-type control group, both AdV@LYO and CSP-AdV@LYO achieved significantly higher intestinal viral colonization, confirming the effective mediation of intestinal targeted delivery by their enteric properties. Among them, AdV@LYO exhibited the highest colonization level. Although the intestinal colonization level of CSP-AdV@LYO was slightly lower than that of AdV@LYO. In contrast, CSP-PL-AdV@LYO showed a lower colonization level in the intestine, indicating that its targeted delivery and protective effects did not meet expectations.

The results of the in vivo toxicity evaluation are shown in Figure 7D. The survival rate of mice in the control group reached 100%, which may be attributed to a large portion of the virus being inactivated by gastric acid during oral administration, paradoxically leading to a higher survival rate. The AdV@LYO group exhibited a high mortality rate of 75%, indicating a significant acute toxic response. Since this formulation did not utilize the sporopollenin carrier for protection, the virus likely underwent rapid burst release in the gastrointestinal tract, resulting in excessively high systemic exposure within a short period and consequently increased mortality. In contrast, CSP-AdV@LYO showed significantly reduced toxicity, with a mortality rate of only 25%. Furthermore, all surviving mice displayed good general clinical conditions, including activity and feeding, during the observation period. This result suggests that the incorporation of the sporopollenin carrier, potentially by modulating the viral release behavior and reducing burst release, significantly improved the preliminary safety profile of the formulation, demonstrating better potential for clinical translation. Finally, the CSP-PL-AdV@LYO group also achieved a 100% survival rate within 24 h. However, the surviving mice exhibited poor health conditions, including lethargy, decreased appetite, hunched posture, and rough fur. This indicates that despite the higher survival rate compared to the CSP-AdV@LYO group, the safety of the phospholipid-coated sample warrants further investigation.

According to the H&E staining results of tissue sections in Figure 7E, in the histological assessment of lung tissue, the negative control group showed minor structural abnormalities. In contrast, the AdV@LYO group presented relatively severe diffuse alveolar damage and inflammatory cell aggregation, indicating that this formulation moderately induced lung pathology. The CSP-AdV@LYO group, modified with chrysanthemum sporopollenin, showed a certain degree of improvement. The extent and severity of inflammatory infiltration were reduced compared to the AdV@LYO group, and the destruction of alveolar structure was noticeably alleviated. This suggests that CSP modification can provide a sustained-release effect, thereby mitigating tissue damage caused by the formulation. The lung tissue morphology of the CSP-PL-AdV@LYO group was close to the normal physiological structure, showing only mild inflammatory reactions, and the alveolar structure remained largely intact. This proves that the composite modification system of CSP and PL can effectively protect lung tissue, significantly reduce the toxic side effects of the formulation, and exhibit the best biocompatibility.

In the intestinal tissue sections, the negative control group showed normal intestinal structure. Both the AdV@LYO and CSP-AdV@LYO groups exhibited obvious intestinal villi shortening, disruption of epithelial integrity, and inflammatory cell infiltration in the lamina propria. These pathological changes are consistent with the specific process of viral colonization in the intestine. The intestinal damage in the CSP-AdV@LYO group was milder than in the AdV@LYO group, suggesting that chrysanthemum sporopollenin encapsulation can provide a certain sustained-release effect for the internal virus, thereby reducing the impact on the intestinal mucosa. The intestinal structure in the CSP-PL-AdV@LYO group recovered to near-normal, indicating that the phospholipid coating made viral release in the intestine difficult, leading to significantly reduced colonization. Furthermore, it shows that the carrier itself is not toxic to the intestine.

In summary, CSP-AdV@LYO achieved the best balance between efficacy and safety.

## 4. Discussion

This study originated from addressing two fundamental challenges in the clinical application of oral delivery of live adenovirus: the extreme instability of the virus itself during in vitro storage and in vivo delivery and the potential sensitization risk associated with traditional pollen carriers. To address the former, inspired by nature’s mechanism of protecting genetic material in pollen for hundreds of millions of years, a biomimetic delivery strategy integrating “exine armor protection, intelligent enteric release, and spike-mediated adhesion” was proposed. To address the latter, this study, through dual screening via proteomic analysis and a zebrafish in vivo allergy model, identified low-allergenic chrysanthemum pollen as the source material. The superior performance of Chrysanthemum sporopollenin originates from its inherent physicochemical properties [21]. Its spike-like nanostructures increase the specific surface area, promoting mechanical interlocking with the intestinal mucus layer. The more negative zeta potential of Chrysanthemum sporopollenin (−31.6 mV) compared to rape sporopollenin (−24.3 mV) is attributed to a higher density of surface carboxyl groups from its unique sporopollenin biosynthesis pathway, which enhances electrostatic repulsion against aggregation and modulates mucin interactions [38]. For the anti-inflammatory activity ranking observed in Figure 2, three key properties contribute: (i) abundant surface carboxyl/phenolic groups for reactive oxygen species scavenging, (ii) the spike morphology for improved mucoadhesion and localized retention, and (iii) optimal particle size for transmucosal diffusion. The entire research followed the main thread of “screening the carrier-constructing the system-validating delivery performance,” ultimately successfully developing the biomimetic oral delivery system for live adenovirus (CSP-AdV@LYO) and systematically verifying its effectiveness and superiority as a novel solution.

The research initially revealed, through proteomic analysis, significant differences in the quantity and spatial distribution of allergenic proteins among different pollens, providing a rational basis for screening. Chrysanthemum and rape pollen showed the lowest levels of allergen proteins across all categories, particularly the fewest strongly allergenic proteins on the surface, indicating low risk at the molecular level. Subsequent functional validation using the zebrafish allergy model confirmed this prediction: the tryptase-like activity in the chrysanthemum and rape pollen treatment groups showed no significant difference from the negative control group. This “screening and verification” process was crucial, ensuring that all subsequent work was based on a raw material with guaranteed biosafety, directly addressing the fundamental obstacle for clinical application of pollen highlighted in the introduction. Acid hydrolysis of these low-allergenic pollens yielded sporopollenin with >90% protein removal as confirmed by elemental analysis, effectively minimizing the allergen burden. Scanning electron microscopy revealed distinct microstructures across sources: the dense spikes and intact hollow cavity of Chrysanthemum sporopollenin laid the physical foundation for its subsequent superior mucoadhesion and drug-loading performance [39,40]. This was not merely a morphological record but also foreshadowed their functional differences: the dense spikes and intact hollow cavity structure of chrysanthemum sporopollenin provided the physical foundation for its subsequent excellent mucoadhesion and drug-loading performance. Particle size and zeta potential analysis indicated improved colloidal stability of the processed sporopollenin, with chrysanthemum sporopollenin exhibiting the most negative potential, favoring its dispersion in aqueous systems and creating conditions for subsequent uniform drug loading. Experiments on macrophage and neutrophil recruitment in zebrafish further confirmed, at the immune cell level, that the deproteinized sporopollenin had significantly lower sensitization potential than the original pollen, with chrysanthemum sporopollenin performing the best. This completed the chain of safety validation from “low-allergenic pollen” to “low-allergenic sporopollenin material.”

After obtaining the safe carrier material, we conducted an in-depth investigation into whether it could achieve the two core design functions: “intelligent enteric release” and “mucosal adhesion.” The analysis of pH responsiveness perfectly revealed the potential of sporopollenin as an intelligent carrier. Chrysanthemum sporopollenin contracted in size with a contact angle as high as 129.4° (strongly hydrophobic) at pH 2.0, while it swelled and the contact angle sharply decreased to 15.5° (strongly hydrophilic) as the pH increased. This reversible “acid-shrinking/alkali-swelling” behavior forms the physicochemical foundation for achieving intestinal targeted release. In the acidic gastric environment, the contracted hydrophobic surface acts like tightly sealed armor, effectively preventing the intrusion of gastric acid and enzymes. Upon entering the neutral intestinal environment, swelling and hydrophilization facilitate the opening of germination pores, promoting the release of the cargo. More importantly, in vitro mucosal retention experiments demonstrated that the model drug loaded in chrysanthemum sporopollenin was cleared from the biomimetic mucosa at a much slower rate than the free drug. In vivo imaging further showed that the sporopollenin carrier significantly prolonged the gastrointestinal retention time of the drug in mice. This aligns perfectly with the strategy proposed in the introduction of utilizing its “spike structures” for mechanical interlocking to enhance mucosal adhesion, offering the possibility for the drug to exert a prolonged local effect in the intestine after oral administration.

Building on this comprehensive understanding of the carrier’s properties, this study proceeded to optimize and construct the final adenovirus delivery system. Optimization experiments on drug loading performance indicated that the vacuum loading method was the most efficient, and the loading capacity increased with rising pH, directly related to the “alkali-swelling” characteristic of sporopollenin. The addition of 0.1% (*w*/*v*) Tween 80 further enhanced loading efficiency: as a non-ionic surfactant, Tween 80 reduces interfacial tension between Chrysanthemum sporopollenin and the aqueous medium, forming a competitive adsorption layer that displaces weakly bound impurities and improves wetting, thereby facilitating drug infiltration into the sporopollenin cavity [41]. We screened a sucrose-gelatin (1:1) formulation as the optimal freeze-drying protectant, which preserved the highest viral DNA copy number, addressing the stability issue during adenovirus lyophilization. Subsequently, the three formulations—AdV@LYO, CSP-AdV@LYO, and CSP-PL-AdV@LYO—were prepared, laying the foundation for subsequent comparative studies.

In vitro stability experiments were key to validating the “exine armor protection” strategy. Under the harsh challenge of simulated gastric fluid, CSP-AdV@LYO released 3-fold higher viral DNA copy numbers compared to AdV@LYO and 20-fold higher compared to free virus. This result strongly demonstrates that the inert hollow cavity structure of chrysanthemum sporopollenin provides superior physical isolation for the virus, protecting it from rapid degradation by gastric acid, which is a prerequisite for successful oral delivery. In the simulated release test, the adenovirus released from capsules at each time point successfully infected susceptible cells and replicated, as evidenced by the detection of progeny viral genomes after six days of culture. This result confirms that the encapsulation and gastrointestinal release process did not compromise the biological infectivity of the adenovirus. Importantly, the preservation of infectivity is a prerequisite for the successful oral delivery of adenoviral vectors, as only infectious virus particles can initiate target gene expression and elicit desired biological effects in vivo. Together with the qPCR detection of viral mRNA in target tissues in vivo, these in vitro infectivity data collectively support that the orally delivered adenovirus retains both transcriptional and infectivity competence, which are essential for its intended application. Such controlled release favors the establishment of appropriate and prolonged local exposure of the virus to the intestinal mucosa. Stability test results (3 months) showed that viral genome copy number retention was most stable in CSP-AdV@LYO, predicting a significantly extended shelf-life. This confirms that sporopollenin not only protects the virus during gastrointestinal transit but also provides a stable microenvironment for long-term storage, addressing the inherent storage and delivery instability of live adenovirus.

The definitive in vivo studies comprehensively evaluated the translational potential of this delivery system from both delivery efficiency and safety dimensions. Viral tissue colonization experiments revealed that CSP-AdV@LYO achieved effective intestinal targeting, with significant viral loads detected in the intestine and minimal distribution in the lungs. This result is crucial, indicating that the sporopollenin carrier not only successfully delivered the virus to the target site but also effectively inhibited systemic dissemination, confining its action locally to the intestinal mucosa. This provides ideal conditions for localized delivery to the intestinal mucosa while reducing systemic toxicity risks. The results of the acute toxicity test presented a stark contrast: the mortality rate in the AdV@LYO group was as high as 75%, while it decreased to 25% in the CSP-AdV@LYO group. Combined with histopathological observations, the AdV@LYO group showed significant pathological damage in intestine, whereas lesions were markedly alleviated in the CSP-AdV@LYO group. This clearly indicates that unprotected virus likely undergoes rapid release in the intestine, causing severe systemic reactions. In contrast, the sporopollenin carrier, through its sustained-release characteristics and targeting effect, substantially improved the safety window. Although CSP-PL-AdV@LYO exhibited the best tissue biocompatibility morphology, its low intestinal colonization efficiency and viral release suggested that the phospholipid coating, while enhancing safety, might have excessively hindered effective viral release. Therefore, further optimization is needed to balance “delivery efficacy” and “safety.”

In this study, total RNA was extracted and viral mRNA was detected rather than directly quantifying viral genomic DNA, based on the following considerations. First, the presence of mRNA reflects active viral gene transcription, which directly demonstrates that the adenoviral vector has successfully reached the target tissues (lungs and intestines) and exhibits biological activity at the transcriptional level. Second, because we performed quantitative PCR (qPCR) using tissue-derived cDNA after reverse transcription, we were able to normalize the samples using a reference gene (e.g., GAPDH), thereby enabling relative quantification and comparison of viral mRNA transcript levels across different treatment groups. This approach allowed us to objectively evaluate the transcriptional efficiency of the vector in target tissues under different doses or administration routes—information that cannot be obtained by merely measuring residual input genomic DNA. Therefore, this method not only confirms targeted delivery of the vector but also provides a more reliable and biologically meaningful quantitative basis for intergroup comparisons.

This study systematically completed a full cycle, from screening low-allergenic raw materials and analyzing carrier functions to constructing an intelligent formulation and conducting comprehensive in vitro and in vivo validation, constituting a complete closed-loop study on pollen sporopollenin. The chrysanthemum sporopollenin carrier is not merely a simple physical encapsulation material. It integrates three functions—“chemically inert armor,” “pH-responsive intelligent switch,” and “spike-enhanced mucosal anchoring”—which work synergistically to overcome the core bottlenecks faced by the oral delivery of live adenovirus: gastric acid inactivation, poor storage stability, inadequate intestinal targeting, and suboptimal safety. The CSP-AdV@LYO delivery system not only significantly enhanced the in vitro storage and in vivo delivery stability of adenovirus but also, through intestinal targeting and sustained-release characteristics, enhanced the potential for mucosal delivery of live adenovirus while markedly reducing systemic toxicity. This truly embodies the strategic advantage of the biomimetic design concept.

It should be noted that the present study primarily focuses on demonstrating the delivery, biodistribution, and persistence of adenoviral material after oral administration, rather than assessing functional immune outcomes. Although these data provide foundational evidence for the feasibility of this platform, we recognize that additional functional endpoints are required to fully establish its biological significance. For instance, studies using the same encapsulated adenovirus platform for vaccine development should evaluate antigen-specific humoral immune responses and neutralizing antibody responses induced by the released virus in immunized animals, which would strongly support the biological relevance and translational potential of this strategy.

With respect to biosafety, we acknowledge that systemic viral exposure, viral dissemination, and long-term translational hurdles were not evaluated in the current study. Since orally administered viruses colonize the intestinal tract, there is a certain risk of viral shedding into the environment. Our data confirm viral mRNA expression in the lungs and intestines, indicating successful delivery and transcriptional activity at these target sites; however, the extent of systemic exposure, potential viral shedding, and long-term safety remain to be determined. Future work will systematically address these limitations to enable a comprehensive safety assessment of this oral adenovirus delivery platform for clinical translation.

Limitations: This study has several limitations that should be acknowledged. First, the proteomic allergen-association screening presented in Figure 1 was a single-run exploratory analysis without biological or technical replicates, so definitive quantitative comparisons among pollen types require further validation with repeated LC-MS/MS runs. Second, the 3-month stability study under 2–8 °C/75% ± 5% RH conditions and the 24 h acute toxicity observation are relatively short; longer-term stability testing and chronic toxicity assessments are needed for clinical translation. Third, the biodistribution analysis was restricted to the intestine and lungs, and potential viral accumulation in other organs (e.g., liver, spleen, kidneys) was not evaluated. Fourth, viral infectivity was confirmed via in vitro infection of HEK293 cells, but functional immune outcomes and long-term in vivo efficacy were not assessed in this study. These limitations will be addressed in future follow-up studies.

## 5. Conclusions

In this study, we successfully developed a biomimetic oral delivery system for live adenovirus based on chrysanthemum sporopollenin, leveraging its “acid-shrinking/alkali-swelling” responsiveness, spike-mediated mucosal adhesion, and chemical inertness. The system improved adenoviral genomic copy number retention by more than 10-fold under the suboptimal 2–8 °C/75% ± 5% RH storage condition and enhanced gastrointestinal mucosal retention by ~3-fold compared to free ICG solution. Critically, the encapsulated adenovirus retained transcriptional activity and infectivity after gastrointestinal transit, supporting the feasibility of this platform for oral adenoviral delivery. While limitations remain regarding long-term stability, systemic safety, and functional immune validation, this work provides a novel, low-allergenic biomimetic strategy for oral delivery of adenovirus and other labile biomacromolecules. Future studies will focus on extending stability testing, evaluating chronic toxicity and immunogenicity, and advancing translational development of this platform.

## Figures and Tables

**Table 1:** Prescription screening of freeze-drying protective agents.

Prescription	A	B	C	D
Sucrose (% *w*/*w*)	0	0	25	25
Gelatin (% *w*/*w*)	0	50	25	0
Phospholipid (% *w*/*w*)	0	0	0	25

## Data Availability

The data presented in this study are available upon request from the corresponding author.
